# Genital herpes shedding episodes associate with altered spatial organization and activation of mucosal immune cells

**DOI:** 10.1172/jci.insight.197491

**Published:** 2025-11-18

**Authors:** Finn MacLean, Rachael M. Zemek, Adino Tesfahun Tsegaye, Jessica B. Graham, Jessica L. Swarts, Sarah C. Vick, Nicole B. Potchen, Irene Cruz Talavera, Lakshmi Warrier, Julien Dubrulle, Lena K. Schroeder, Anna Elz, David Sowerby, Ayumi Saito, Katherine K. Thomas, Matthias Mack, Joshua T. Schiffer, R. Scott McClelland, Keith R. Jerome, Bhavna H. Chohan, Kenneth Ngure, Nelly Rwamba Mugo, Evan W. Newell, Jairam R. Lingappa, Jennifer M. Lund, Kinga Study Team

**Affiliations:** 1 Vaccine and Infectious Disease Division, Fred Hutchinson Cancer Center, Seattle, Washington, USA.; 2 Department of Global Health, University of Washington, Seattle, Washington, USA.; 3 Cellular Imaging Shared Resource, and; 4 Fred Hutch Innovation Lab, Fred Hutchinson Cancer Center, Seattle, Washington, USA.; 5 Department of Internal Medicine-Nephrology, University Hospital Regensburg, Regensburg, Germany.; 6 Department of Medicine and; 7 Department of Laboratory Medicine and Pathology, University of Washington, Seattle, Washington, USA.; 8 School of Public Health, Jomo Kenyatta University of Agriculture and Technology, Nairobi, Kenya.; 9 Center for Clinical Research, Kenya Medical Research Institute, Nairobi, Kenya.; 10 Department of Pediatrics, University of Washington, Seattle, Washington, USA.; 11 See Supplemental Acknowledgments for details on the Kinga Study Team.

**Keywords:** Immunology, Infectious disease, Cellular immune response

## Abstract

Herpes Simplex Virus 2 (HSV-2) infection results in variable rates of local viral shedding in anogenital skin. The effect of episodic viral exposures on immune cells in adjacent mucosal tissues, including the genital tract, is unknown. However, any immune responses at this site could affect protective mucosal immunity, tissue homeostasis, and adverse health outcomes. To investigate the effect of HSV-2 on cervicovaginal tract immunity, we applied flow cytometry, immunofluorescence imaging, analysis of soluble immune factors, and spatial transcriptomics to cervicovaginal tissue and blood samples provided by a total of 232 HSV-2–seropositive and seronegative participants, with genital HSV-2 shedding evaluated at the time of biopsy. This unique dataset was used to define and spatially map immune cell subsets and localized gene expression via spatial transcriptomics. HSV-2 seropositivity alone was associated with minimal differences in cervicovaginal and circulating T cell phenotypes. However, the vaginal mucosa during active HSV-2 shedding was associated with alterations in T cell, macrophage, and DC localization and gene expression, consistent with increased immune surveillance, with immune activating and suppressing signals potentially reinforcing mucosal tissue homeostasis.

## Abstract 2

In context of episodic HSV-2 shedding, immune cells mobilize and co-localize in the vaginal epithelium, expressing cytotoxic and inflammatory plus immunoregulatory genes that collectively may promote tissue homeostasis to limit damage.

## Introduction

Immunologists have classically divided infection into 2 discrete categories — acute and chronic. Acute infections, such as influenza virus, norovirus, and pertussis, tend to resolve within days or weeks. In contrast, chronic infections, including Hepatitis C virus, tuberculosis, HIV-1, and CMV, are characterized by infection persisting over months to years, with persistent exposure of the host to pathogen-derived antigen plus inflammation. Studies of chronic infections have revealed adaptations in immune responses to restrict immunopathology in settings of host response to persistent antigen exposure, including the development of T cell exhaustion (1). Rapid microbial clearance during acute infection, however, leaves the host with functional immune memory that can be recalled upon subsequent exposure to the same infection. Infection by some notable pathogens of public health significance do not fit this dichotomy but, rather, exhibit episodic infection, wherein exposure to antigen and/or inflammation is occasional or frequent but not persistent. This includes infection with human herpes viruses including HSV-2.

HSV-2 is a highly prevalent and lifelong infection. Following initial infection of the genital skin, HSV-2 ascends through neurites via retrograde transport and establishes latency most commonly in peripheral neuronal ganglia. Cycles of viral latency and reactivation can result in episodic release of virus either in the context of painful genital ulcers, fissures, or more commonly through asymptomatic viral shedding in the genital skin and mucosa (2). HSV-2 is a leading cause of genital ulcer disease (GUD), with a recent estimate of 178 million people worldwide suffering from at least 1 episode of HSV-2–related GUD in 2016, or nearly 5% of the global population of 15- to 49-year-olds (3). This tremendous burden of disease underscores the need to develop improved prevention tools, including therapeutic vaccines, which requires an enhanced understanding of natural immunity to the virus at the sites of viral exposure. Here, we characterize immune cells present in distinct anatomic sites in HSV-2–seropositive and HSV-2–seronegative individuals, as well as a subset of individuals actively shedding HSV-2 virus.

Detailed studies of HSV-2 shedding have revealed that genital shedding rates, though somewhat variable among individuals, are highest during the first year of infection and then stabilize over subsequent years (4–6). Genital sampling at 6-hour intervals for 2 months demonstrated that shedding occurred a median of 18 times per year in individuals seropositive for HSV-2 (HSV-2–seropositive individuals), although most episodes are cleared in less than 24 hours (7). This extremely rapid viral clearance following genital shedding suggests successful tissue immunity. Mathematical modeling indicates that a surprisingly low density of HSV-specific tissue-resident T cells is sufficient for repeated effective local elimination of HSV-2–infected cells (6, 8–11). Furthermore, skin from HSV-2–infected individuals reveals clusters of HSV-specific CD4^+^ and CD8^+^ T cells (12–16) and Treg (17) at sites of HSV-2 lesions and after lesional healing. More recently, we demonstrated transient changes in tissue T cells in HSV-2 lesions, although overall, the number of skin T cells and markers of activation and function were stably maintained within genital skin tissues in response to episodes of HSV-2 reactivation (18). Studies of a mouse model of HSV infection have clarified features of this protective tissue-resident memory T cell (T_RM_) response upon HSV challenge (19, 20). Notably, using parabiosis combined with a mouse model of vaginal HSV-2 infection, Iijima and Iwasaki demonstrated that CD4^+^ T_RM_ establish tissue residency in memory lymphocyte clusters within the vaginal mucosa and were crucial for viral containment upon secondary HSV-2 exposure. Furthermore, chemokine production by local macrophages maintained these T_RM_ (20), thus highlighting the importance of tissue immune cell organization in achieving viral control in the context of shedding. Finally, murine studies and mathematical models demonstrate that a higher density of T_RM_ within reactivation microenvironments predicts more rapid infected cell elimination and lower viral loads (8, 9, 21, 22).

A notable difference between most murine and human studies of anti-HSV-2 immunity is the anatomic site of study. Mouse models of HSV-2 have focused on immune cells in the vagina (2), whereas many human HSV-2 studies have tracked immune cells within genital skin. CCR5^+^ CD4^+^ T cells persist after HSV-2 lesion healing in human genital skin (13), thereby suggesting a potential immune-based mechanism to explain the observed increased risk of HIV-1 acquisition in HSV-2–seropositive individuals (23). Assessment of tissue T cells in the genital mucosa of HSV-2–seropositive individuals may provide additional insight, as the genital mucosa including the vaginal tract (VT) and ectocervix (CX) are more likely sites of sexual HIV-1 acquisition (24). A limited number of human studies of the female genital tract mucosa have demonstrated that HSV-2–specific T cells are present in the CX of HSV-2–seropositive individuals (25–29), though immune cells in the vagina have not been assessed.

We have previously characterized T cells within the human CX and VT and identified distinct activation profiles across genital tract sites compared with cells within the circulation (30–33). Given the remaining gaps in our knowledge of how HSV-2 shedding affects immunity in female genital tract tissues, with implications for sexual transmission of HIV-1 and other sexually transmitted infections, here we comprehensively assess not only cellular profiles associated with HSV-2 infection but also the immune cell localization, organization, and gene expression in the vaginal tissue during episodes of HSV-2 viral shedding.

## Results

### Study population characteristics.

In total, 232 participants (*n* = 135 HSV-2–seronegative and *n* = 97 HSV-2–seropositive) from the Kinga Study met the criteria to be included in this study. HSV-2–seropositive individuals were mostly asymptomatic at the time of sample collection, with comparable rates of sores reported at any time in the 3 months prior to their visit (4.4% HSV-2–seronegative versus 6.2% HSV-2–seropositive) and with no participants self-reported to be taking HSV medication at their enrollment visit. At enrollment, 2 of 135 seronegative individuals reported a history of GUD in the past 3 months, and of 94 seropositive participants with a response to the question, none reported a history of GUD in the previous 3 months. This low prevalence of GUD is consistent with other studies of individuals in Africa (34). Study participants were young (87% younger than 40), with the HSV-2–seronegative group being relatively younger than the HSV-2–seropositive group (Table 1), likely due to fewer lifetime exposures to HSV-2 in the younger, HSV-2–seronegative group. This cohort was sexually active (median sex acts of 8 per month) in generally monogamous relationships, with 41.7% of all females using hormonal contraceptives. Of note, 32.0% of HSV-2–seropositive individuals (versus 8.1% of HSV-2–seronegative individuals) had a sexual partner living with HIV (and remained HIV-1/2 seronegative throughout this study) (Table 1).

To limit the effect of potentially confounding variables on local cervicovaginal tract (CVT) immunology analyses, we adjusted flow cytometry analysis of the CX and VT tissue samples, as well as soluble immune factor analysis from CVT fluid, for the following variables: age (continuous variable), bacterial vaginosis (BV) status diagnosed by Nugent score (categories defined in Table 1), HIV exposure (defined as having their heterosexual partner living with HIV), hormonal contraceptive use (categories defined in Table 1), and semen exposure (continuous variable defined as sex acts per month). We adjusted systemic immune analyses, including PBMC flow cytometry analysis and serum soluble immune factor analysis, for age and hormonal contraceptive use to limit the effect of potential confounding variables.

### HSV-2 seropositivity is not associated with a shift in the proportion of T cell subsets or T cell density in the VT or CX.

**Figure 1:**
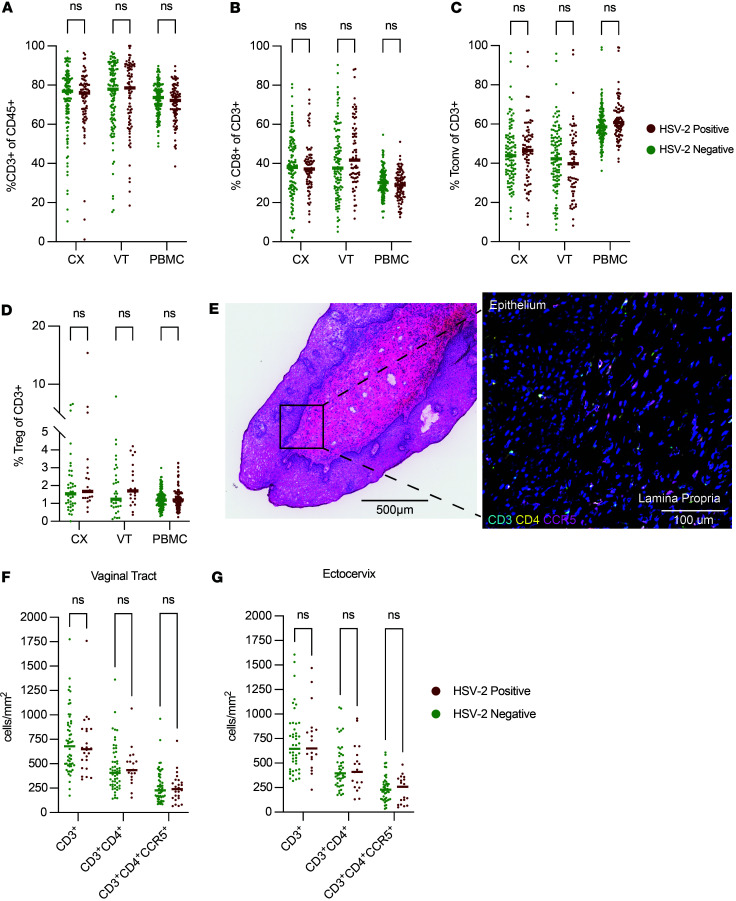
HSV-2 seropositivity not associated with alterations in total T cells or subsets in the genital mucosa or circulation. (**A**) Flow cytometry was used to measure the proportion of CD3^+^ cells among total CD45^+^ cells in ectocervix (CX), vaginal tract (VT), and PBMC samples. (**B**–**D**) CD8^+^ frequency (**B**), CD4^+^ CD25^–^CD127^+^/^–^ conventional T Cell (Tconv) (**C**), and CD4^+^CD25^+^CD127^–^Foxp3^+^ Treg among total CD3^+^ T cells in CX, VT, and PBMC (**D**). (**E**) Representative H&E image of VT tissue (left), and representative immunofluorescence-stained VT tissue section from the same sample as shown in the H&E image (right). The box represents the portion of the serial section that is shown in the immunofluorescence image. Scale bars 500 µm or 100 µm, as indicated on image. (**F** and **G**) Quantification of the density of CD3^+^, CD3^+^CD4^+^, and CD3^+^CD4^+^CCR5^+^ cells in VT (**F**) and CX (**G**) tissue sections. Each dot represents an individual sample, and each bar represents the median. For flow cytometry analysis, comparisons were made using an adjusted rank regression model. PBMC comparisons were adjusted for hormonal contraceptive use and age, and CX and VT comparisons were adjusted for hormonal contraceptive use, bacterial vaginosis via Nugent score, HIV exposure, semen exposure, and age. Image analysis was done on a subset of samples that were unexposed to HIV and samples that were BV^–^. Wilcoxon rank sum test was used to compare cellular densities via image analysis without adjustment. “ns” indicates results were nonsignificant with an adjusted *P* value (for flow cytometry) and an unadjusted *P* value (for image analysis) greater than 0.05.

Given the predominance of CD3^+^ T cells among total CD45^+^ immune cells in the CVT and blood (Figure 1A) and the potential importance of HSV-2–mediated T cell responses to local viral shedding, we focused our flow cytometry–based analysis on major T cell subsets and their phenotypes (Supplemental Table 1; supplemental material available online with this article; https://doi.org/10.1172/jci.insight.197491DS1). We analyzed the proportion of CD8^+^ T cells (Figure 1B), conventional CD4^+^ T cells (Tconv) (Figure 1C), and Treg (Figure 1D) as a fraction of total T cells across tissue sites comparing HSV-2–seropositive versus –seronegative individuals. After adjustments, we found no statistically significant alterations by HSV-2 serostatus in the proportions of CD8^+^ T cells, Tconv, or Treg in any of the 3 tissue sites. Additionally, we quantified the numbers and location of T cells in the VT and CX through microscopy. Cryopreserved VT and CX tissue biopsies were sectioned and stained to assess the density and location of CD3^+^ total T cells and CD3^+^CD4^+^ T cells (Figure 1E). As with flow cytometry, we found no differences in the density of CD3^+^ or CD3^+^CD4^+^ cells in the VT or the CX when assessed as a whole (Figure 1, F and G) or when analyzing the epithelium and lamina propria of each tissue separately (Supplemental Table 2).

An adverse outcome associated with HSV-2 infection is increased HIV susceptibility (23). Previous studies have observed increased potential HIV target cells expressing the HIV coreceptors CCR5^+^ and CD4^+^ on T cells in the healing HSV-2 skin lesion (13) and in CVT cytobrushes from HSV-2–seropositive versus HSV-2–seronegative individuals (35, 36) without assessing whether this phenomenon is true in the deeper CVT tissue layers of those with HSV-2. We hypothesized that greater abundance of CCR5^+^ activated CD4^+^ T cells in the CVT, where sexual exposure to HIV likely occurs, may account for the documented epidemiologic association of increased HIV susceptibility among those HSV-2–seropositive. Therefore, we also assessed CD3^+^CD4^+^CCR5^+^ cell density in the VT and CX and found no differences in either tissue site when assessed as a whole (Figure 1, F and G) or when analyzing the epithelium and lamina propria separately (Supplemental Table 2). In total, we demonstrate that asymptomatic HSV-2 seropositivity is not significantly associated with alterations in the overall populations of major immune cell subsets in CX, VT, or PBMC samples, nor does it affect T cell and HIV target cell density within the CX or VT tissue layers.

### Vaginal T cells from HSV-2–seropositive individuals displayed altered expression of markers associated with intrinsic and extrinsic immunoregulation.

**Figure 2:**
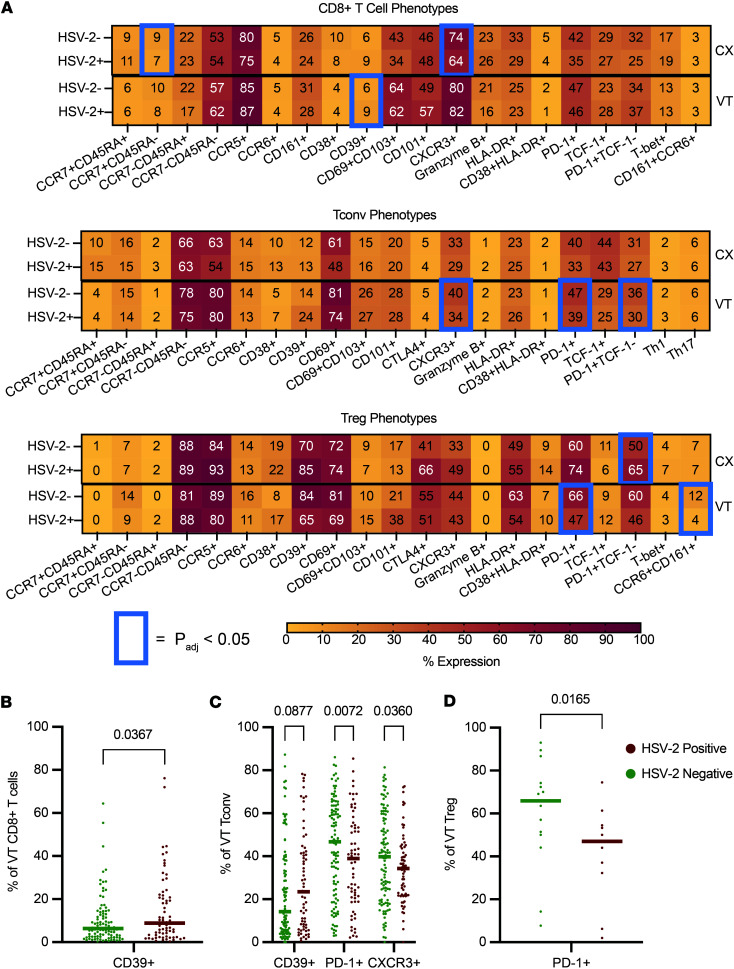
HSV-2 seropositivity drives few phenotypic alterations in the CVT. (**A**) Heatmap showing frequency of CD8^+^ T cell, CD4^+^CD25^–^CD127^+^/^–^ Tconv, and CD4^+^CD25^+^CD127^–^Foxp3^+^ Treg phenotypes in the CX and VT among HSV-2–seropositive and –seronegative individuals. All comparisons were made using a rank regression model with adjustments as in Figure 1. Comparisons on the heatmap that have an adjusted *P* < 0.05 are boxed in blue. (**B**) Frequency of CD8^+^ T cells expressing CD39 in the VT of HSV-2–seropositive versus –seronegative individuals. (**C**) Frequency of Tconv expressing CD39, PD-1, or CXCR3 in the VT of HSV-2–seropositive versus –seronegative individuals. (**D**) Frequency of Treg expressing PD-1 in the VT of HSV-2–seropositive versus –seronegative participants. Each dot represents an individual sample, and each bar represents the median. Adjusted rank regression *P* values are displayed. *N* is the same in **B**–**D** as described in **A**. For *n* values see Table 1.

Given the proximity of the VT to probable sites of viral reactivation in the anogenital skin, we hypothesized that HSV-2 seropositivity would correlate with phenotypic alterations of T cell subsets in the VT. Thus, we also evaluated the expression of Tbet to identify Th1 type Tconv cells (Th1) and CD161 and CCR6 double positivity to identify Th17 type Tconv cells (Th17), plus many phenotypic markers following an experimental design previously described (33). We looked broadly for differences in activation and other phenotypic markers in CD8^+^ T cells, Tconv, and Treg in the CX and VT (Figure 2A), as well as in Th1 and Th17 cells (Supplemental Table 1). We found a significant increase in the fraction of CD8^+^ T cells that express CD39 in the VT of HSV-2–seropositive individuals (median HSV-2–seropositive = 9% versus HSV-2–seronegative = 6%; adjusted rank regression β = 2.61, *P*_adj_ = 0.0367) (Figure 2B). Additionally, the proportion of CD4^+^ Tconv cells in the VT that express CD39 was increased in HSV-2–seropositive individuals (median HSV-2–seropositive = 24% versus HSV-2–seronegative = 14%; adjusted rank regression β = 4.89; *P*_adj_ = 0.0844), but did not reach our nominal threshold (*P*_adj_<0.05) for statistical significance (Figure 2C). CD39 expression was also increased, but failed to meet the nominal significance threshold, on CX CD8^+^ T cells (median HSV-2–seropositive = 9% versus HSV-2–seronegative = 6%; adjusted rank regression β = 2.16; *P*_adj_ = 0.0877) with relatively equivalent CD39 expression observed on CX Tconv (median HSV-2–seropositive = 13% versus HSV-2–seronegative = 12%; adjusted rank regression β = 1.09; *P*_adj_ = 0.6250) (Figure 2A and Supplemental Table 1).

In addition to altered CD39 expression, we found a significant reduction in the proportion of VT Tconv and Treg that express PD-1 in HSV-2–seropositive individuals (median Tconv HSV-2–seropositive = 39% versus HSV-2–seronegative = 47%; adjusted rank regression β = –11.48; *P*_adj_ = 0.0072; median Treg HSV-2–seropositive = 47% versus HSV-2–seronegative = 66%; adjusted rank regression β = –27.14; *P*_adj_ = 0.0165) (Figure 2, C and D) with a reduced effect observed on CX Tconv (median HSV-2–seropositive = 33% versus HSV-2–seronegative = 40%; adjusted rank regression β = –4.00; *P*_adj_ = 0.2927) and a marginal, nonsignificant increase observed on Tregs (median HSV-2–seropositive = 74% versus HSV-2–seronegative = 60%; adjusted rank regression β = 15.98; *P*_adj_ = 0.1788) (Figure 2A and Supplemental Table 1). Lastly, we observed a significant decrease in the fraction of Tconv cells that express the chemokine receptor CXCR3 in the VT of HSV-2–seropositive individuals (median HSV-2–seropositive = 34% versus HSV-2–seronegative = 40%; adjusted rank regression β = –7.90; *P*_adj_ = 0.0360) (Figure 2C). In sum, by applying flow cytometry to CVT biopsies, we identified a limited number of VT T cell phenotypic alterations associated with HSV-2 seropositivity, with fewer significant differences identified in the CX.

### Circulating T cells are primed for trafficking while also displaying signs of regulation in HSV-2–seropositive individuals.

**Figure 3:**
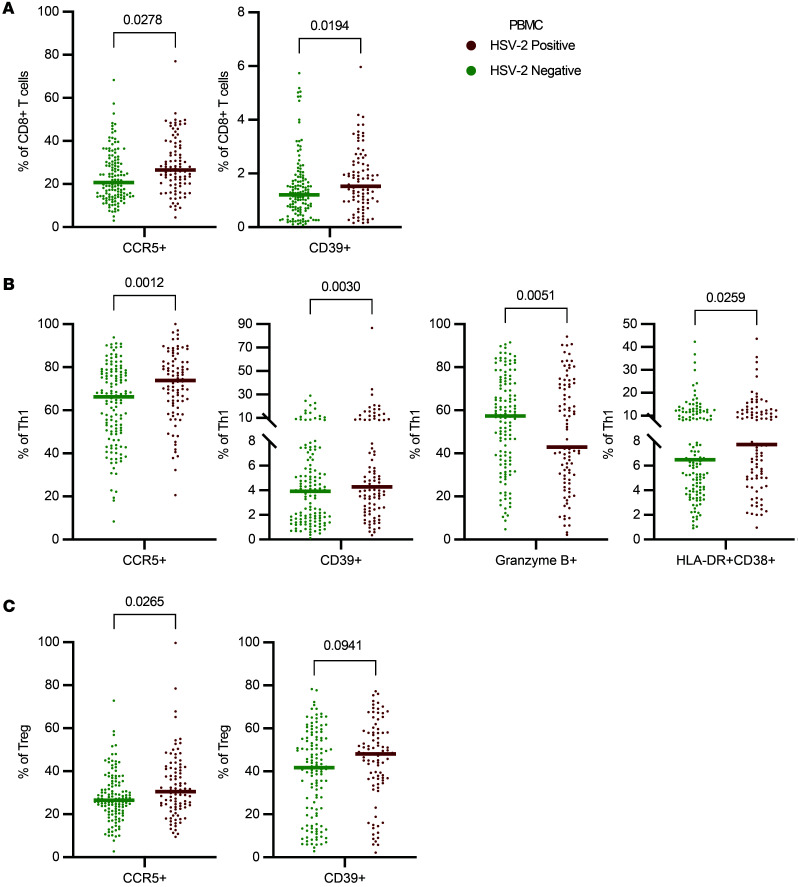
HSV-2 seropositivity is associated with circulating T cell signatures. (**A**) The frequency of CD8^+^ T cells in PBMC samples from HSV-2–seropositive versus –seronegative participants that express CCR5 or CD39. (**B**) The frequency of Th1 cells (Tbet^+^Tconv) in PBMC samples from HSV-2–seropositive versus –seronegative participants that express CCR5, CD39, Granzyme B, or HLA-DR/CD38 double positivity. (**C**) The frequency of Treg expressing CCR5 or CD39 in PBMC samples from HSV-2–seropositive versus –seronegative participants. PBMC comparisons were made using a rank regression model that adjusted for hormonal contraceptive use and age. The adjusted *P* value is displayed on each graph. Each dot represents an individual data point, and each bar represents the median for that group.

We next evaluated whether HSV-2 was associated with signatures of altered phenotypes in circulating T cells (Supplemental Table 1 and Supplemental Figure 1). Our analysis revealed that HSV-2–seropositive individuals had an increased proportion of CCR5^+^CD8^+^ T cells (median HSV-2–seropositive = 27% versus HSV-2–seronegative = 21%; adjusted rank regression β = 3.49; *P*_adj_ = 0.0278) (Figure 3A) and CCR5^+^ Th1 cells (median HSV-2–seropositive = 74% versus HSV-2–seronegative = 66%; adjusted rank regression β = 8.47; *P*_adj_ = 0.0012) (Figure 3B) in the circulation, which may have the potential to traffic to distal anatomic sites and/or contribute to inflammatory antiviral immune responses. Circulating Tregs from HSV-2–seropositive individuals also more frequently expressed CCR5 (median HSV-2–seropositive = 31% versus HSV-2–seronegative = 27%; adjusted rank regression β = 3.50; *P*_adj_ = 0.0265) (Figure 3C).

CD39 was also significantly more frequently expressed by circulating CD8^+^ T cells (median HSV-2–seropositive = 1.53% versus HSV-2–seronegative = 1.21%; adjusted rank regression β = 0.32; *P*_adj_ = 0.0194) (Figure 3A) and Th1 cells (median HSV-2–seropositive = 4.3% versus HSV-2–seronegative = 3.9%; adjusted rank regression β = 1.23; *P*_adj_ = 0.0030) (Figure 3B). Additionally, there was a trend toward an increase in the fraction of Tregs expressing CD39 (median HSV-2–seropositive = 48% versus HSV-2–seronegative = 42%; adjusted rank regression β = 4.56; *P*_adj_ = 0.0941) (Figure 3C). Treg expression of CD39 is associated with greater antiinflammatory potential and could contribute to Treg-dependent immunoregulation in the context of recurrent antiviral immune responses (37).

Finally, Th1 from the PBMC samples of HSV-2–seropositive individuals display reduced expression of the cytotoxic effector molecule Granzyme B (median HSV-2–seropositive = 43% versus HSV-2–seronegative = 57%; adjusted rank regression β = –9.58; *P*_adj_ = 0.0051), despite having a greater level of activation as measured by CD38^+^HLA-DR^+^ expression (median HSV-2–seropositive = 7.7% versus HSV-2–seronegative = 6.5%; adjusted rank regression β = 1.42; *P*_adj_ = 0.0259) (Figure 3B). In all, HSV-2 infection may alter systemic T cell phenotypes and drive a systemic signature of differential T cell activation. This signature may reflect a balancing act driven by extrinsic and intrinsic regulatory mechanisms to retain homeostasis and prevent immunopathology while maintaining the potential to readily traffic and respond to viral reactivation in mucosal sites.

### Several soluble immune factors are decreased in serum from HSV-2–seropositive individuals.

In total, 71 soluble immune factors were analyzed by Eve Technologies Human Cytokine Array/Chemokine Array from the serum and CVT fluid (Softcup) to assess the cytokine and chemokine milieu in circulation and the genital mucosa. For soluble factors with any values falling out of the linear range of the assay, these values were imputed (see Supplemental Methods), and the resulting data analyzed as a continuous variable. If > 20% of data were imputed, then the data were analyzed as a dichotomous variable of detected versus not detected. In the serum, 53 cytokines/chemokines were quantified as a continuous outcome (Supplemental Figure 2A and Supplemental Table 3), and in the CVT fluid, 61 cytokines/chemokines were quantified as a continuous outcome (Supplemental Figure 3A and Supplemental Table 3). In the serum, 16 factors were analyzed as dichotomous outcomes (Supplemental Figure 2B and Supplemental Table 3), with 2 serum soluble immune factors excluded for being greater than the detectable range for more than 20% of samples and therefore without variation when treated as detectable versus not detectable. Ten factors from CVT fluid were analyzed as dichotomous outcomes (Supplemental Figure 3B and Supplemental Table 3). These analyses identified significant differences in 9 serum factors: PDGF-AA, MIP-1β, Fractalkine, MCP-3, VEGF-A, TNF-α, TNF-β, IL-13, IL-4 (all reduced), and no significant (*P*_adj_ < 0.05) alterations when comparing HSV-2–seropositive versus –seronegative CVT fluid samples.

### Spatial transcriptomics identifies tissue and immune cell subsets within VT tissue.

**Figure 4:**
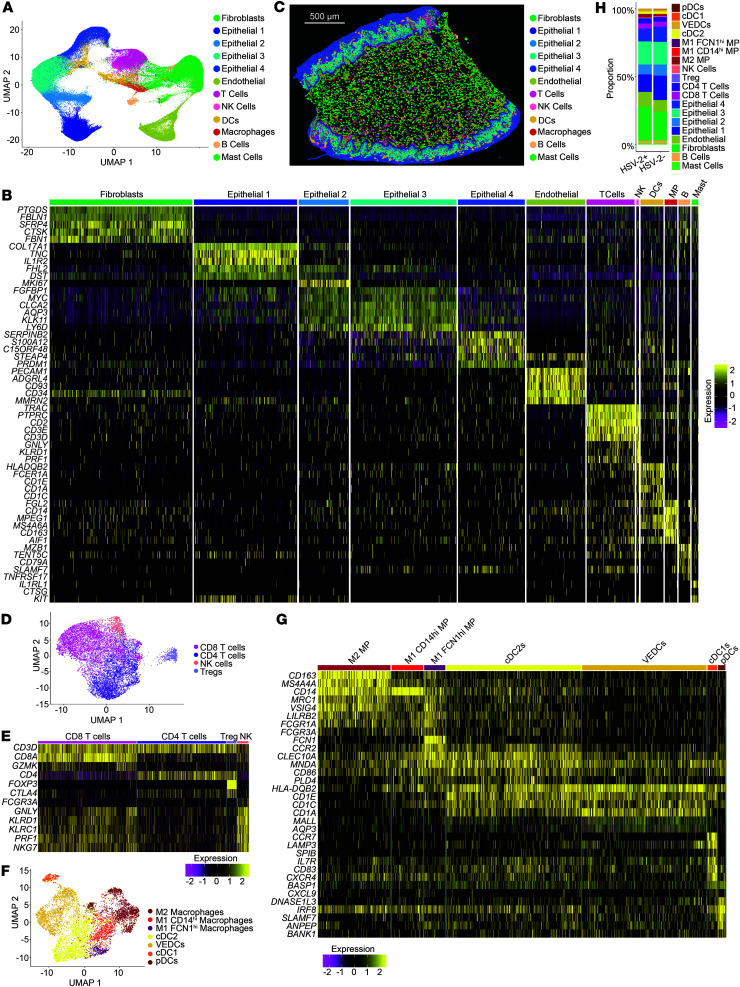
Spatial Transcriptomics identifies cell types and location in the tissue. (**A**) Annotated UMAP showing the clustering of cells pooled from all samples analyzed on the Xenium platform. (**B**) Heatmap showing distinguishing transcripts used to identify cell types. (**C**) The visualization of cells on a representative tissue section. Scale bar: 500 µm.(**D**) UMAP used to distinguish T cell subsets and NK cells from the broader T cell cluster. (**E**) Heatmap showing transcripts used to distinguish subsets in **D**. (**F**) UMAP used to distinguish innate immune cell subsets from the DC and macrophage (MP) clusters in **A**. (**G**) Heatmap showing transcripts used to distinguish subsets in **F**. (**H**) Comparisons of cell types present in the tissue between *n* = 5 HSV-2–seropositive and *n* = 6 HSV-2–seronegative individuals.

To assess immune alterations associated with HSV-2 seropositivity plus active HSV-2 shedding and their position within tissue layers more deeply, we used the 10x Genomics Xenium platform to analyze the location and transcriptional profile of immune cells in VT tissue sections from 5 HSV-2–seropositive and 6 HSV-2–seronegative Kinga Study participants (Table 2). Spatial transcriptomics was performed on vaginal tissue, as this is the anatomic region within the genital mucosa where we identified differences in T cell phenotypes in HSV-2–seropositive individuals (Figure 2A). We also reasoned that HSV-2 shedding from the anogenital skin was more likely to access vaginal tissue due to spatial proximity. We applied 10x Xenium v1 Spatial gene expression using the Human Multi-Tissue and Cancer panel, consisting of 377 genes. Cell segmentation was performed using Proseg (38), which uses cell morphologies identified from DAPI-stained nuclei and the spatial distribution of transcripts to determine cell boundaries. Low-quality cells (< 10 probe counts and < 5 features) were removed, leaving a total of 142,903 cells. From the panel, we identified 4 epithelial layers, fibroblasts, endothelial cells, and immune cell types (Figure 4, A and B), which mapped to the anatomically expected areas and structures (Figure 4C and Supplemental Figure 4A). The 4 epithelial clusters are mapped to the inner layer (stratum basalis) (Epithelial 1), 2 suprabasal layers (Epithelial 2 and 3), and an outer layer (stratum corneum) (Epithelial 4).

The T cell/NK cell cluster was subclustered to identify CD8^+^, CD4^+^, Treg, and NK cells (Figure 4, D and E). The macrophage and DC clusters were subclustered to identify subtypes including M1-like macrophages (*CD14*^hi^ and *FCN1*^hi^ populations), M2-like macrophages (*CD163*^+^*MRC1*^+^), conventional DC-2 (cDC2) populations (*CD1C*^+^*CD1A*^+^), cDC1 (*CCR7*^+^*LAMP3*^+^), and plasmacytoid DCs (pDC) (*IRF8*^hi^*SLAMF7*^+^*ANPEP*^+^) (Figure 4, F and G). The 2 cDC2 populations distinguish themselves as a more cDC2-like population (*CD1C* and *CLEC10A*^hi^), and the other similar to a vaginal epithelial DC (VEDC) phenotype (*CD1A*^hi^), which resides in the outer stratum corneum layer.

After identifying cellular populations in the vaginal tissue sections, we compared the cellular composition in samples from HSV-2–seropositive and HSV-2–seronegative individuals. Similar to what we detected by flow cytometry for T cell categories (Figure 1), we found no significant difference in the proportion of any cell type between HSV-2–seropositive and HSV-2–seronegative samples (Figure 4H and Supplemental Figure 4B). Altogether, through use of spatial transcriptomics, classified a comprehensive range of tissue types, stratifying the mucosal layers, submucosa and endothelial cells in vaginal tissue and defining subsets of immune cell types.

### VT samples from participants with active HSV-2 shedding contain more immune cells with an inflammatory phenotype.

While we were able to identify select T cell phenotypes in the genital mucosa and circulation that associate with HSV-2 seropositivity, we predicted that active viral shedding may have a more pronounced effect on the local genital immune cell localization. HSV-2–seropositive participants provided an anogenital swab at the time of tissue biopsy collection. Three participants had detectable HSV-2 virus, confirming they were actively shedding HSV-2 virus at the time of biopsy collection (Shed^+^), and 2 HSV-2–seropositive participants had a negative anogenital PCR sample and served as the comparator group (Shed^–^). Given the small N included in our spatial transcriptomics analysis, no statistical adjustments were made for potentially confounding variables. Each participant included in this analysis was unexposed to HIV, and there were no participants who were known to be BV^+^, though some clinical data are missing from individual participants. One HSV-2 Shed^+^ participant reported having sores on their genital area in the previous 3 months but none at the time of biopsy collection, and none of the others reported sores on their genital area at their visit or any time in the last 3 months.

**Figure 5:**
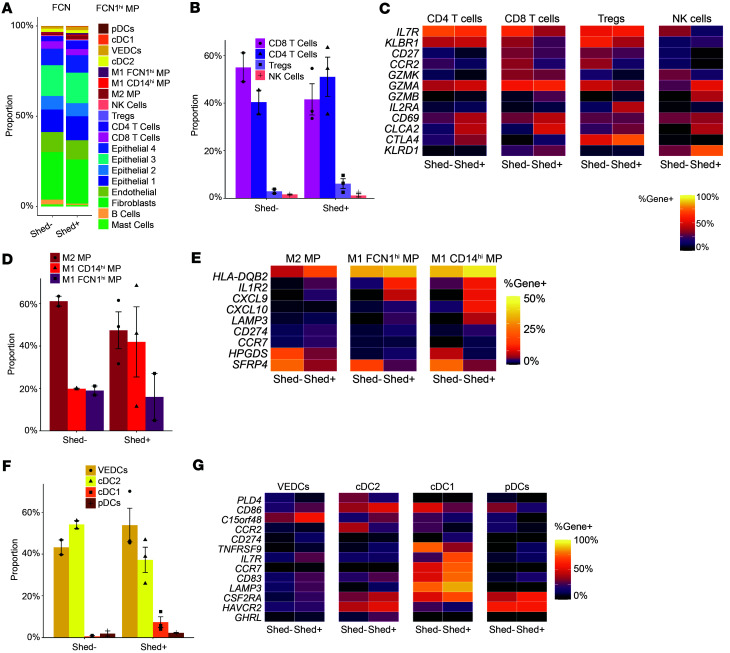
HSV-2 viral shedding associates with inflammatory immune response in the vagina. (**A**) Proportion of cell subsets in HSV-2–seropositive, anogenital swab HSV-2–PCR negative (Shed^–^) (*n* = 2) versus HSV-2–seropositive, anogenital swab HSV-2 PCR positive (Shed^+^) (*n* = 3). (**B**) Comparison of T cell subsets and NK cells as the frequency of total T and NK cells. (**C**) Heatmap showing relative gene expression in T and NK cell subsets in Shed^–^ versus Shed^+^ samples. (**D**) Comparison of macrophage (MP) subsets as the frequency of total cells in the macrophage cluster. (**E**) Heatmap showing relative gene expression in macrophage subsets in Shed^–^ versus Shed^+^ samples. (**F**) Comparison of DC subsets as the frequency of total cells in the DC cluster. (**G**) Heatmap showing relative gene expression in DC subsets in Shed^–^ versus Shed^+^ samples.

Because we hypothesized that active HSV-2 anogenital shedding may have distinct effects on vaginal immune cells, we compared participants who were actively shedding HSV-2 virus to those with a negative anogenital swab PCR result. Within the T cell compartment, Shed^+^ samples showed a shift from CD8^+^ to CD4^+^ dominant, with a nonsignificant increase in Treg (Figure 5, A and B). In terms of cellular profiles, Shed^+^ individuals had a lower percentage of CD4^+^ and CD8^+^ T cells that expressed *CD27*, *CCR2*, and *GZMK*, and a higher percentage that expressed *CD69*, *CLCA2*, and *CTLA4*. CD8^+^ T cells also displayed increased expression of *GZMB* in Shed^+^ samples, suggesting a shift to an activated, cytotoxic phenotype. A greater percentage of Tregs expressed *IL2RA* and *CTLA4* in Shed^+^ samples, required for Treg survival and suppressive function. NK cells were also more activated in the Shed^+^ samples, with a greater percentage expressing *GZMA*, *GZMB*, and *KLRD1* (Figure 5C and Supplemental Figure 5A). To complement our Xenium analysis, we examined T cell phenotypes in vaginal and cervical biopsies and PBMC from HSV-2–seropositive individuals in our flow cytometry analysis based on HSV-2 shedding status. Among the very few differences identified were an increase in the fraction of CD4^+^ Tconv in the vagina that were CD38^+^ in HSV shedders compared with nonshedders (Supplemental Figure 4C), along with a decrease in the fraction of cervical CD8^+^ T cells that had an exhausted phenotype (PD-1^+^ or PD-1^+^TCF-1^–^) or a central memory phenotype (CCR7^+^CD45RA^–^; Supplemental Figure 4D).

We also assessed the effect of HSV-2 shedding on macrophage subsets. Shed^–^ samples had predominantly M2 macrophages, while Shed^+^ samples had a greater proportion of M1 macrophages, particularly the CD14^hi^ M1 population (Figure 5D). These M2 macrophages expressed *HPGDS* and *SFRP4* in the Shed^–^ samples, indicative of a wound-healing or antiinflammatory phenotype. In Shed^+^ samples, a greater percentage of M1 macrophages, particularly CD14^hi^ M1 macrophages, expressed *IL1R2*, *CXCL9*, *CXCL10*, and *LAMP3*, genes known to be upregulated in inflammatory settings (39–41) (Figure 5E and Supplemental Figure 5B).

Similarly, in the DC compartment, Shed^+^ samples showed a shift from cDC2 (*CD1C*^+^ *CLEC10A*^+^) to cDC1 (*CCR7*^+^*LAMP3*^+^*CD83*^+^), with significantly more cDC1 cells in Shed^+^ samples (Figure 5F). Notably, a greater percentage of cDC1 expressed *CD83* and *LAMP3* in individuals with active shedding, possibly indicating increased maturity and activation in response to viral stimuli. Furthermore, increased *CCR7* expression in cDC1 of Shed^+^ samples may indicate increased DC maturation and ability to migrate toward the draining LN (Figure 5G and Supplemental Figure 5C). The cDC1 cells also expressed more *IL7R* and *CSF2RA*, suggesting they may be migratory or monocyte derived. In Shed^+^ samples, more CD1a^hi^ VEDC expressed *C15ORF48*, potentially indicative of an inflammatory response (42). In contrast, Shed^–^ samples had more DCs with *CCR2* expression, suggestive of an immature phenotype (Figure 5G and Supplemental Figure 4C). Altogether, analysis of immune cell subsets and gene expression by different types of immune cells in the vagina of HSV-2–seropositive individuals indicates that active HSV-2 shedding is associated with modest shifts in composition of the immune cell compartment and changes toward more inflammatory gene expression.

### Active HSV-2 shedding is associated with macrophage polarization and the recruitment of CD4^+^ T cells into the epithelium.

**Figure 6:**
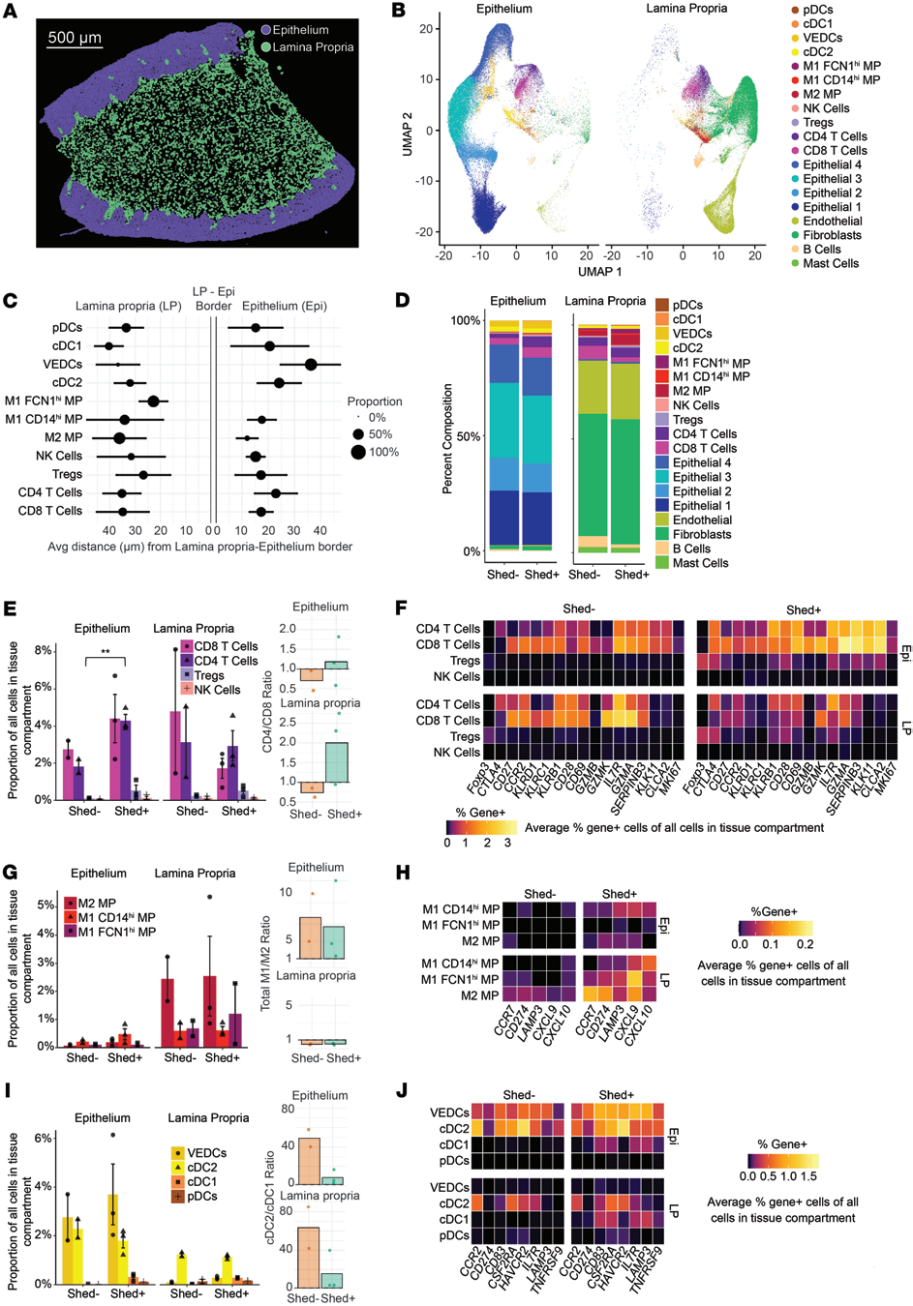
Vaginal inflammatory immune response is enhanced in the epithelium during HSV-2 shedding. (**A** and **B**) BuildNicheAssay function in Seurat was used to stratify the epithelium (Epi) and lamina propria (LP) layers, and clustering cells in each layer confirmed differences in cell populations in each layer. (**A**)Scale bar: 500 µm. (**C**) The average distance and distribution of each cell type from the thin basal epithelium cell layer were measured. (**D**) The proportion of cell types in the lamina propria and epithelium in Shed^–^ versus Shed^+^ samples was measured. (**E** and **F**) The proportion of T cell subsets and NK cells in Shed^–^ versus Shed^+^ (*n* [Shed^–^] = 2, *n* [Shed^+^] = 3) (**E**), and the heatmap comparing gene expression in T and NK cells in Shed^–^ versus Shed^+^ (**F**). (**G** and **H**) The proportion of macrophage subset (MP) in Shed^–^ versus Shed^+^ (**G**), and the heatmap comparing gene expression in macrophage subsets in Shed^–^ versus Shed^+^ (**H**). (**I** and **J**) The proportion of DC subsets in Shed^–^ versus Shed^+^ (**I**), and the heatmap comparing gene expression in DC subsets in Shed^–^ versus Shed^+^ (**J**). **P < 0.01, calculated using a nonparametric permutation test, with Benjamini-Hochberg correction for multiple comparisons.

Given the alteration in immune cells and genes associated with cell migration or recruitment, we hypothesized the location of immune cells between the mucosa and submucosa may be altered during active viral shedding. To explore the spatial distribution of immune cells, the BuildNicheAssay function in the Seurat package was adapted to distinguish between the lamina propria and epithelium (Figure 6, A and B). Not only did cell types differ in their location between the 2 niches, but also their distance from the basal epithelial layer, with FCN1^hi^ M1 macrophages closest to the basal layer within the lamina propria, CD14^hi^ M1 macrophages venturing into the epithelial layer, and VEDCs farthest out into the epithelium. T cell populations were found in both the lamina propria and epithelium, although CD4^+^ T cells on average ventured the furthest distance into the epithelium (Figure 6C). Comparison of the proportion of cells in each niche revealed that Shed^–^ samples had a higher proportion of T cells in the lamina propria, while Shed^+^ samples had more T cells in the epithelium and more macrophages in the lamina propria (Figure 6, D–G). This suggests that HSV-2 shedding is associated with differences in the composition of the immune cell compartment within the vaginal tissue layers.

When further comparing cells within each niche, we found significantly more CD4^+^ T cells within the epithelium in Shed^+^ samples compared with Shed^–^, but no significant difference in the lamina propria (Figure 6E). This was confirmed by calculating the density of T cells (cells per μm^2^), where the density of CD4^+^ T cells was increased in the epithelium in Shed^+^ samples, though results were nonsignificant (Supplemental Figure 6A). Immunofluorescence staining confirmed an increase, albeit not statistically significant, in the CD3^+^CD4^+^ density in the epithelium relative to total CD3^+^ density in the vaginal tissue (Supplemental Figure 6B), confirming our transcript-level findings. Comparing the CD4/CD8 T cell ratio, T cells in Shed^–^ samples were predominantly CD8^+^ in both niches, while CD4^+^ cells were the dominant T cell population in both niches in Shed^+^ samples (Figure 6E). In terms of gene expression (Supplemental Figure 6C), Shed^–^ samples were characterized by more CD4^+^ and CD8^+^ T cells in the lamina propria expressing *CD27*, *CCR2*, *KLRB1*, and *IL7R*, consistent with a resting memory phenotype (43). In contrast, this population is diminished in the Shed^+^ samples, which have more *CD28*, *CD69*, *GZMA*, *KLK11*, and *CLCA2* expression in CD4^+^ and CD8^+^ T cells in the epithelium (Figure 6F), suggestive of cytotoxic activation. The CD8^+^ T cells in the epithelium of Shed^+^ samples express the genes for granzymes *GZMB*^+^, *GZMK*^+^, and *GZMA*^+^, effector molecules involved in cytotoxicity of virally infected cells (Figure 6F). Additionally, there is increased expression of *CTLA4*^+^ on T cells in the epithelium of Shed^+^ samples, consistent with a regulatory mechanism (Figure 6F). This change in gene expression pattern by T cells within the spatial niches, with more inflammatory T cells residing in the lamina propria in Shed^–^ individuals and more cytotoxic T cells and activated CD4^+^ T cells shifting to the epithelial niche in Shed^+^, may reflect changes in pathogen-associated molecular pattern (PAMP) exposure within the vaginal lumen and vaginal epithelium that could thereby activate innate immune cells and induce changes in cytokine and chemokine expression.

We next explored the distribution and gene expression characteristics of innate, antigen-presenting cell (APC) subsets in the lamina propria and epithelium. Within the macrophage subsets, there was no significant change in the proportions (Figure 6G) or density (Supplemental Figure 6D) of macrophages in the lamina propria regardless of HSV-2 shedding status. There was also no change in the M1/M2 ratio between Shed^–^ and Shed^+^, although the location influenced this ratio, with M2 macrophages dominant in the lamina propria and M1 macrophages dominant in the epithelium. Inflammatory genes, including *CCR7*, *CD274*, *LAMP3*, *CXCL9*, and *CXCL10*, were increased in macrophages within the lamina propria from Shed^+^ samples compared with Shed^–^ (Figure 6H). Shed^+^ samples also had more *LAMP3*, *CXCL9*, and *CXCL10*^+^ macrophages in the epithelial layer, which corresponded to CD14^hi^ M1 macrophages (Figure 6H). The CD14^hi^ M1 were the main source of *CXCL10*^+^ and *CXCL9*^+^ macrophages in the epithelium, which was the macrophage subtype that was not only in both the lamina propria and epithelium but was also furthest into the epithelial layers (Figure 6C). Finally, we identified an inverse correlation between macrophage *CXCL10*/*CXCL9* expression and distance from the basal epithelium, particularly in the epithelium in Shed^+^ samples (*CXCL1*0 *r* = –0.68, FDR = 6.6^–44^, CXCL9 *r* = –0.52, FDR = 4.9^–35^), with most highly expressing cells within 20 μm (Supplemental Figure 6, E and F). Taken together, this is suggestive that macrophages, particularly the activated CD14^hi^ M1 subset, may be a source of CXCL10 and CXCL9 chemokines in Shed^+^ samples, which could in turn attract immune cells such as T cells toward the basal epithelium.

Lastly, within the DC subtypes, Shed^+^ samples had a small yet distinct cDC1 population in the lamina propria and epithelial layers that was not present in Shed^–^ samples (Figure 6I). In addition to *LAMP3* and *CCR7*, this population expressed *CD83*, *CSF2RA*, and *IL7R*, suggestive of an inflammatory migratory population (Figure 6J). There was also a slight decrease in the cDC2 population in Shed^+^ samples, resulting in a decrease in the cDC2/cDC1 ratio (Figure 6I), though there was no difference in total DC density across experimental groups (Supplemental Figure 6G). The cDC2 population showed a shift from *CCR2*^+^ in Shed^–^ to *LAMP3*^+^ in Shed^+^ (Figure 6J), indicative of maturation. Although there was no change in the number of VEDCs associated with active viral shedding, they showed the strongest shift in gene expression, with an increase in cells in the epithelium expressing genes associated with IFN stimulation, such as *CD274*, *HAVCR2*, and *TNFRSF9*, known to be upregulated during active viral shedding (Figure 6J). Altogether, we show that, in the context of active HSV-2 shedding, there are changes in the composition of the immune cells in both the lamina propria and the epithelium, and there is increased expression of activation and inflammatory genes within the epithelial layer in Shed^+^ samples. This is consistent with mobilization of cells, possibly mediated at least in part through CXCL9/CXCL10 (Supplemental Figure 6, E and F), that are involved in immune surveillance and effector function to an anatomic site that may be exposed to viral shedding (the vaginal lumen).

### Inflammatory macrophages may draw T cells toward the epithelium, where they interact with DCs during active HSV-2 shedding.

**Figure 7:**
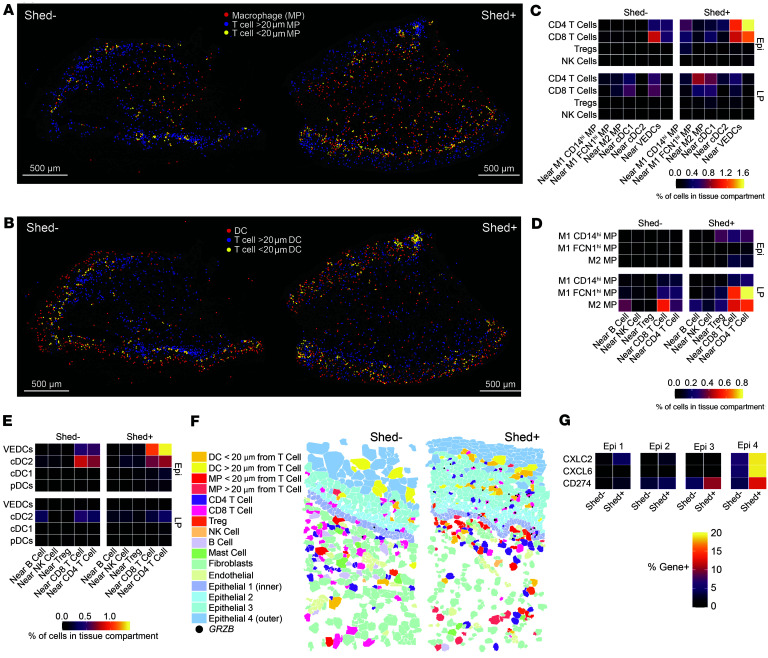
HSV-2 shedding promotes cellular interactions in vaginal epithelium. (**A**) Representative image of a Shed- and Shed+ VT tissue section highlighting T cells farther than 20 µm away from macrophages (purple) and T cells near macrophages (yellow), with macrophages shown in red. (**B**) Representative image of a Shed- and Shed+ VT tissue section highlighting T cells father than 20 µm away from DCs (purple) and T cells near DCs (yellow), with DCs shown in red. (**C**) Heatmap showing the proportion of T cells within 20 µm of select antigen presenting cells.(**D** and **E**) Heatmap showing the proportion of macrophages (**D**) and DC types (**E**) within 20 μm of T cells stratified by subsets and NK cells. (**F**) Representative portion of a tissue section from a Shed^–^ and a Shed^+^ sample to demonstrate cellular interactions that are more frequently observed in Shed^+^ samples. (**G**) Heatmap showing *CXCL2*, *CXCL6*, and *CD274* gene expression by epithelial (Epi) subtype in Shed^–^ and Shed^+^ samples.

We sought to identify cellular interactions within different tissue regions to better understand the coordinated networks of immunity to active viral shedding. To look for cell interactions, distances between cells were calculated, with a distance of 20 μm used as a cutoff for cells directly interacting with one another (Supplemental Methods). Using this metric, cells could be classified as near (<20 μm) or far (>20 μm) from a target cell of interest, followed by querying the frequency of interactions between T cells and APCs by tissue compartment (Figure 7, A and B, and Supplemental Figure 7, A–C). We first examined T cell subset colocalization with APCs and found that most interactions with macrophages occurred in the lamina propria (Figure 7C and Supplemental Figure 7, D and E), predominantly at the edge of the basal epithelium in Shed^+^ samples (visual representation; Figure 7A). T cells interacting with DCs were located mostly in the epithelial layer (Figure 7C and Supplemental Figure 7, F and G); however, in Shed^–^ samples, most interactions occurred near the basal epithelium, including in the lamina propria, while in Shed^+^ samples, most of the interactions were throughout the epithelium (Figure 7B). In Shed^–^ samples, CD8^+^ and CD4^+^ T cells mainly interacted with cDC2 or M2 macrophages in the lamina propria and cDC2 or VEDCs in the epithelium, with the most abundant interaction between cDC2 and CD8^+^ T cells (Figure 7C). In Shed^+^ samples, CD4^+^ T cells interacted with cDC1 and FCN1^hi^ M1 macrophages in the lamina propria, and cDC1 in the epithelium, CD4^+^, CD8^+^, and Treg interacted with CD14^hi^ M1 macrophages in the epithelium; both CD4^+^and CD8^+^ T cells had more interactions with cDC1 and VEDCs in the epithelium (Figure 7C).

These findings were confirmed by examining macrophage interaction partners. M2 macrophages predominantly interacted with CD8^+^ T cells in the lamina propria in Shed^–^ samples (Figure 7D and Supplemental Figure 7, H and I). In Shed^+^ samples, most interactions were between FCN1^hi^ M1 macrophages and CD4^+^ T cells in the lamina propria (Figure 7D). While there were very few macrophage interactions in the epithelium of Shed^–^ samples, there were interactions between M2 and, particularly, CD14^hi^ M1 macrophages and CD4^+^, CD8^+^, and Treg in the epithelium of Shed^+^ samples (Figure 7D). These data suggest that, during HSV-2 shedding, there are slight increases in T cell interactions, particularly CD4^+^ T cell interactions with M1 macrophage populations in the lamina propria, and that uniquely in the context of active viral shedding, macrophage–T cell crosstalk also occurs in the epithelial layer.

We also looked at DC and T cell interactions and found that cDC2 was the dominant subtype that interacted with both CD4^+^ and CD8^+^ T cells in Shed^–^ samples (Figure 7E and Supplemental Figure 7, J and K). In samples from individuals with active HSV-2 shedding, however, this shifts to a larger fraction of VEDC interacting with CD4^+^ T and CD8^+^ T cells (Figure 7E). This suggests that, in Shed^+^ samples, T cells are being drawn toward VEDCs, which reside in the outer layers of the epithelium.

Through visual examination of spatial interactions, we observe that, in the Shed^–^ sample, fewer macrophages are interacting with T cells in the lamina propria, with some CD8^+^ T cells in the epithelial layer, but not far enough to interact with the VEDCs in the outer epithelium (Figure 7F). In contrast, in the Shed^+^ sample, we observe macrophages interacting with CD4^+^, CD8^+^, and Treg close to the basal epithelium, with more CD4^+^ and CD8^+^ T cells in the epithelium interacting with DCs, particularly further toward the outer layers (Figure 7F). This supports our interpretation that, during active viral shedding, T cells are being drawn from the lamina propria toward the basal epithelium and interacting with macrophages, before being drawn into the outer vaginal epithelium, where they are interacting with VEDCs that express genes associated with maturation and activation.

Finally, we investigated the vaginal epithelial cell compartment for differences in gene expression relevant to immunity to HSV-2 shedding. An analysis of the epithelial cells found that a greater proportion of the outer epithelial cells expressed *CXCL2*, *CXCL6*, and *CD274* in the Shed^+^ samples (Figure 7G and Supplemental Figure 7L). *CD274*, or PD-L1, is known to be upregulated when stimulated by IFN in response to viral infection (44), providing support that viral-immune interactions are occurring in the outer epithelium of the VT during active HSV2 shedding. These inflammatory cues induced by epithelial cells in conjunction with immune cells responding to an active viral infection could drive the mobilization and activation of the DC and macrophages, which may, in turn, play a role in the recruitment and activation of T cells. Altogether, our characterization of immune cell organization and gene expression in the vaginal lamina propria and epithelium in HSV-2–seropositive individuals reveals that active viral shedding associates with changes in immunity within the genital mucosa. Notably, we observed changes in macrophage, DC, and T cell mobilization toward the epithelial layers, and increased expression of genes related to inflammation, cytotoxicity, activation, and intrinsic regulation.

## Discussion

Previous studies investigating immune response dynamics against HSV-2 reactivation have understandably focused on genital lesions, where viral infection and reactivation are known to occur. Studies have also used cytobrushes, lavage, and other minimally invasive techniques to sample en masse and analyze cervicovaginal immune cells from patients with HSV-2 (27, 36). Here, we characterize HSV-2–driven immune changes in VT and CX tissue biopsies not at a site of ulceration among a cohort of 232 Kenyan women. We also applied recent innovations in spatial transcriptomics to characterize immune changes associated with episodes of viral shedding identified in a largely asymptomatic cohort. While we did not see shifts in major T cell subsets in circulation or tissue compartments comparing people who were HSV-2^+^ to HSV-2^–^ (Figure 1), we did observe altered T cell phenotypes in VT with minimal differences in CX T cell subsets analyzed (Figure 2). Among vaginal T cell differences, we observed greater CD39 expression on T cells in HSV-2–seropositive individuals (Figure 2, B and C). Expression of CD39 is associated with metabolic stress, exhaustion, and immune dysfunction (45). In contrast, we observed a reduction in PD-1 expression on VT T cells in HSV-2–seropositive individuals (Figure 2C), as well as in HSV shedders compared with nonshedders (Supplemental Figure 4D). Both PD-1 and CD39 are associated with immune exhaustion (46), so opposing expression patterns in association with HSV-2 seropositivity or shedding is surprising. However, it’s possible that CD39 indicates chronic T cell stimulation, as has been seen in tumor contexts (47), rather than exhaustion.

We hypothesized that HSV-2 seropositivity would have the greatest effect on altering genital immune responses and were, thus, surprised to find it associated with more differences in the circulating immune signature. A key marker defining this immune signature was increased CCR5 expression on Th1, CD8^+^ T cells, and Tregs. CCR5-expressing T cells have been well documented to play a critical role in tissue-specific responses, as the chemokine receptor that can help draw T cells into barrier tissue sites, and elevated CCR5 expression on circulating immune cells may allow them to more readily home to sites of viral reactivation. Mouse models have shown that, during HSV-2 reexposure, macrophages produce CCL5, the ligand for CCR5, thereby leading to the recruitment and retention of CCR5^–^expressing T cells in the mucosa (20). While we do not observe an increased frequency of CCR5 expression in the genital tract mucosal tissue, circulating CCR5-expressing memory T cells may support genital skin-specific immune responses, where CCR5-expressing cells have been identified (13).

In addition to the VT immune signature that we identified in HSV-2–seropositive participants, we also observed signs of intrinsic and extrinsic regulation on circulating T cells. Although the frequency of CD39 expression on T cells is overall lower in the blood than in the tissue (Figures 2 and 3), expression was significantly increased on circulating Th1 and CD8^+^ T cells in HSV-2–seropositive individuals, possibly indicating metabolic stress and/or antigen exposure. Increased CCR5 expression and a modest but insignificant increase in CD39 expression on circulating Tregs may promote Treg activation and greater ability to mediate immunoregulation. While we observed a greater frequency of activated, double-positive CD38^+^HLA-DR^+^ Th1 cells, we observed a reduction in their cytotoxic capacity via reduced granzyme B expression in HSV-2–seropositive individuals (Figure 3B). Reduced Granzyme B expression, along with the reduction of several circulating proinflammatory cytokines (Supplemental Figure 2A), could indicate a degree of immunosuppression or disease tolerance.

We hypothesized that the acute and transient nature of active HSV-2 shedding from the genital skin may lead to immune activation in the neighboring VT and be responsible for altering the spatial organization of cells. Consistent with our flow cytometry analysis, spatial transcriptomics revealed no broad differences in the immune cell proportions between HSV-2–seropositive versus HSV-2–seronegative samples, though viral shedding associated with differences among immune cell subsets and gene expression patterns. When comparing the overall tissue, T cells had more detectable *CD69*, an activation marker promoting tissue retention, and *CLCA2*, which may also aid tissue retention, specifically by elevating E-cadherin binding in the epithelium (48). Additionally, Shed^+^ samples had a trend toward a larger Treg population and more *CTLA4* and *IL2R* expression on Tregs, which supports Treg suppressive function and cell survival in the tissue. The findings suggest that vaginal T cells may adapt to recurrent viral shedding by promoting regulatory phenotypes that enhance disease tolerance (49–51) during HSV-2 shedding. We also observed more M1 proinflammatory macrophages with a CD14^hi^ phenotype in Shed^+^ individuals. A greater proportion of these macrophages expressed *CXCL9* and *CXCL10*, which can promote T cell recruitment to the tissue. Additionally, in Shed^+^ samples, we observed significantly more proinflammatory DC with a cDC1 phenotype that express *CCR7*, *LAMP3*, and *CD83*. *LAMP3* is upregulated in vaginal epithelium during HSV-2 infection (52), which provides further support that these innate immune cells are likely interacting with HSV-2 virus in the vaginal tissue. We also identified VEDCs that resided in the outer epithelial layer and express *C15ORF48*, which was upregulated in Shed^+^ samples. This gene is elevated in response to inflammatory signals and helps to regulate mitochondrial function when oxidative stress occurs, negatively regulating the immune response (53). The switch to proinflammatory phenotypes and upregulation of inflammatory genes is consistent with viral antigen exposure in the VT during HSV-2 shedding. Furthermore, upregulation of regulatory immune phenotypes and gene signatures of immune regulation all suggest that immune regulatory mechanisms are also activated in the context of repeated HSV-2 antigen exposure.

Due to the heterogeneity in tissue layers captured by biopsy sampling, interpretation of the true biology may be obscured. Leveraging the utility of spatial transcriptomics, we performed niche-specific analysis and assessed colocalization of immune cells, leading to greater insights. HSV-2 shedding was associated with increased *CXCL2* and *CXCL6* transcript expression in the outer epithelial cells, and with greater immune activation of macrophages and DCs in the epithelial layer, where we postulate virus-immune cell interactions and proinflammatory immune responses most likely occur in Shed^+^ samples. Shed^+^ samples had more M1-like macrophages, possibly derived from recruited monocytes due to their expression of monocyte associated genes *FCN1* and *CD14* (54, 55). While FCN1 macrophages were located in the lamina propria, close to the basal epithelium, CD14^hi^ macrophages were increased in the epithelium in Shed^+^ samples, adding to the inflammatory profile within the epithelium. Additionally, cells found farthest into the epithelium and closest to the vaginal lumen, including cDC1s, VEDCs, and CD14^hi^ M1 macrophages, had upregulation of proinflammatory and maturation genes, which may indicate encounters with viral antigen.

In concordance with the increased proinflammatory genes in the epithelium, we also observed CD4^+^ and CD8^+^ T cell recruitment into the epithelial layer in Shed^+^ samples. In Shed^–^ samples, the CD4^+^ and CD8^+^ T cells resided in the lamina propria and displayed a resting memory phenotype, including expression of *KLRB1* and *CD27*, which are expressed by T cells that are not active but primed for recall. We hypothesize that these cells are likely antigen experienced from previous active shedding events and are retained in the tissue, poised for recall. In contrast, T cells in Shed^+^ samples appeared to migrate to the epithelium, where they expressed genes associated with cytotoxicity and activation including granzymes such as *GZMB*. The migration and phenotype change in Shed^+^ samples is evidenced by a loss of T cells with a resting memory phenotype in the lamina propria coinciding with the increase of activated T cells in the epithelium. It should also be noted that, while this shift was evident for CD8^+^ T cells, there was an additional increase in CD4^+^ T cells with low expression of the proliferation gene *MKI67*, suggesting that these cells have been recruited, possibly from the blood where we saw significantly more CCR5 expression on CD4^+^ T cells in HSV-seropositive people (Figure 3B). Notably, this increased localization and activation of CD4^+^ T cell in the vaginal epithelium with active HSV-2 viral shedding may increase VT HIV target cells and HIV susceptibility.

We next used spatial data to identify innate and adaptive cell interactions. We found that T cells in Shed^+^ were much more likely to be found in proximity to macrophages and DCs (Figure 7C). Interestingly, macrophage chemokine production was concentrated near the basal epithelial layer (Supplemental Figure 6, E and F), where most macrophages were located and most T cell macrophage interactions occurred in Shed^+^ samples, suggesting a role in T cell recruitment in response to viral antigen. Interestingly, in Shed^+^ samples, *CTLA4* expression was elevated on T cells within the epithelium, and Tregs were increased and preferred to localize near the basal epithelium where they interacted with macrophages, signifying a regulatory response to the inflammation. In contrast, CD4^+^ and CD8^+^ T cells colocalized with DCs further out in the epithelium. Although these T cells were more activated, the DCs and macrophages in the outer epithelium of Shed^+^ samples had increased expression of *CD274*, the gene for PD-L1, which is upregulated in response to interferon and acts as an extrinsic and intrinsic immune regulator (56). Of note, CD4 colocalization with VEDCs was the prominent feature of Shed^+^ samples. VEDCs expressed the costimulatory receptor *TNFRSF9* (41BB/CD137), which promotes T cell proliferation, survival, and cytokine production, as well as the immune checkpoints *CD274* (PDL1) (57) and *HAVCR2* (TIM3) (58, 59), which negatively regulate T cells, perhaps countering excessive inflammation.

These findings reveal that, while proinflammatory immune response may be vital to limiting prolonged viral activation, immunoregulatory networks are likely crucial to balance the tissue response and potentially limit disease pathology to promote tissue homeostasis. Overall, the shifts that we identified in T cell phenotypes associated with HSV-2 seropositivity and active shedding appear to reflect a balance between inflammation and immune regulation. These phenotypes appear to be amplified during episodes of acute mucosal immune activation associated with HSV-2 viral shedding, concurrent with immunoregulatory signals. This regulatory control is likely to be critical during episodic infection, given the “sensing and alarm function” of T_RM_ (60). As T cells migrate into the epithelial layers of the vaginal mucosa in response to local viral shedding, engagement with their cognate antigen may initiate a robust immune cascade at the tissue site, including bystander activation. In such a setting, immune regulatory mechanisms are essential to preserve tissue integrity and prevent immunopathology.

Our study has several limitations. First, our analysis of T cell populations via flow cytometry had limited power to evaluate phenotypic differences in the rarer cell populations due to a limited number of cells isolated from each mucosal biopsy. Additionally, albeit large, our phenotyping antibody panel was limited, leaving many markers unevaluated. Our analysis of tissue sections via immunofluorescence staining and cellular imaging was limited to only 3 markers and a limited number of samples that were of sufficient quality for analysis. Furthermore, there was not sufficient tissue left after the Xenium analysis for validation of findings by immunofluorescence-based approaches. Future studies with new samples would be informative to assess HSV specificity of T cells in cervicovaginal tissues. While our spatial transcriptomics allowed for subcellular resolution, it is a probe-based assay that evaluated 377 individual mRNA transcripts, with only 121 immune-related genes to define cells and phenotypes, leaving many key genes unchecked. While Xenium does provide good sensitivity, it cannot give true gene counts due to the probe-based design, and all samples provided for analysis were small, which introduces potential bias. Lastly, our clinical data were limited to 2 clinical visits. PCR testing for HSV-2 shedding was only done on a subset of HSV-2–seropositive participants at 1 visit, leaving the frequency and history of HSV-2 shedding in the cohort unknown.

In sum, our spatial transcriptomics analysis revealed that the localization, gene expression, and interactions of immune cells are profoundly altered by HSV-2 viral shedding. The vaginal tissue response to viral shedding may be dependent not only on T cell recruitment and cytotoxic activation, but also on regulatory mechanisms including expression of intrinsic immune checkpoint factors to balance a memory tissue response that can quickly contain viral shedding episodes while also reducing the occurrence of symptomatic herpes infection and adverse health outcomes associated with HSV-2 infection.

## Methods

### Sex as a biological variable.

This study focuses on cervicovaginal immune alterations in the context of HSV-2. Therefore, samples from individuals who were identified as female at birth are exclusively used.

### Participants, samples, and data collection.

Samples for this analysis were provided by a subset of participants from the Kinga Study (Clinicaltrials.gov; NCT03701802). For more information about the study and laboratory methods, including microscopy, flow cytometry, cytokine and chemokine analysis, and spatial transcriptomics analysis, see the Supplemental Methods.

### Statistics.

We compared CD3^+^, CD3^+^CD4^+^, and CD3^+^CD4^+^CCR5^+^ cell densities from immunofluorescence imaging experiments between HSV-2–seropositive versus –seronegative participants using the Wilcoxon rank sum test. For flow cytometry experiments, we used rank-based regression, a nonparametric method robust to outliers (61), to compare the percentage of specific T cell phenotypes. For soluble immune factor analysis, we first determined the proportion of total samples that were at least 80% quantifiable for each cytokine/chemokine measured. For those cytokines/chemokines with at least 80% quantifiable detection, we imputed out-of-range values by randomly selecting a value between the lowest observed value and half the lowest observed value (if out-of-range low), or by selecting the largest observed value (if out-of-range high). We then estimated differences in mean log cytokine/chemokine concentrations from both serum and CVT fluid using linear regression. If fewer than 80% of samples were quantifiable, then we categorized each value as either detected or undetected. Next, we estimated the odds ratio for the effect of HSV-2 on the detection of those given cytokines/chemokines that were <80% detectable using logistic regression.

For flow cytometry and soluble immune factor analysis of genital tract samples (VT and CX biopsy and CVT fluid), results were adjusted for hormonal contraceptive use (yes, no and menstruating, no and amenorrheal, or unknown for a small number of samples), bacterial vaginosis (Nugent score: 0–3, negative; 4–6, intermediate; 7–10, positive; or unknown for a small number of samples), HIV exposure (HIV status of sexual partner), number of unprotected sex acts in the last 30 days (continuous), and age (continuous variable). Results from PBMC and serum samples were adjusted for hormonal contraceptive use and age only.

This is an exploratory analysis; we did not adjust our results for multiple comparisons. We considered all nominal *P* values less than or equal to 0.05 as significant. Statistical analysis was done using R version 4.3.3.

### Study approval.

All participants provided written informed consent using documents reviewed and approved by the University of Washington IRB and the Scientific and Ethics Review Unit of the Kenya Medical Research Institute.

### Data availability.

All supporting data are included in the Supplemental figures, tables, the Supporting Data File and/or are available upon reasonable request to the senior authors. The spatial transcriptomics data generated has been deposited in GEO GSE307895. The custom R code for spatial analyses are available at github.com/rachael-z/Kinga_Xenium_HSV_analysis (commit ID ac5d7aa880ec92e4de0469d943c15db3b1284073).

## Author contributions

FM, JBG, JLS, SCV, NBP, ICT, LW, and DS conducted the experiments. FM, RMZ, ATT, JLS, AS, and KKT analyzed data. MM provided reagents. RMZ, JD, LKS, and KRJ contributed analysis methods. BHC, KN, NRM, JRL, and JML designed the research study. JRL, KKT, AE, EWN, JML, and NRM provided supervision. FM, RMZ, JTS, RSM, EWN, JRL, and JML wrote the first draft of the manuscript. All authors edited and approved the manuscript. Co–first authors FM and RMZ are ordered alphabetically.

Funding support

This work is the result of NIH funding, in whole or in part, and is subject to the NIH Public Access Policy. Through acceptance of this federal funding, the NIH has been given a right to make the work publicly available in PubMed Central.

NIH grants R01 AI131914 (to JML and JRL), R01 AI141435 (to JML), R01 HD114505 (to RSM and JML), and R01 AI129715 (to JRL).

T32 AI007509 (SV).

T32 AI007140 (ICT).

T32 AI083203 (LW).

## Supplementary Material

Supplemental data

Supplemental table 1

Supplemental table 2

Supplemental table 3

Supporting data values

## Figures and Tables

**Table 1:**
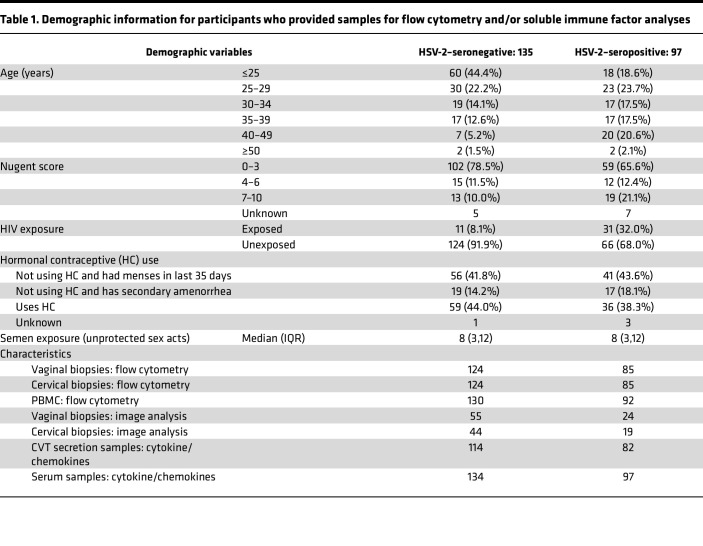
Demographic information for participants who provided samples for flow cytometry and/or soluble immune factor analyses

**Table 2:**
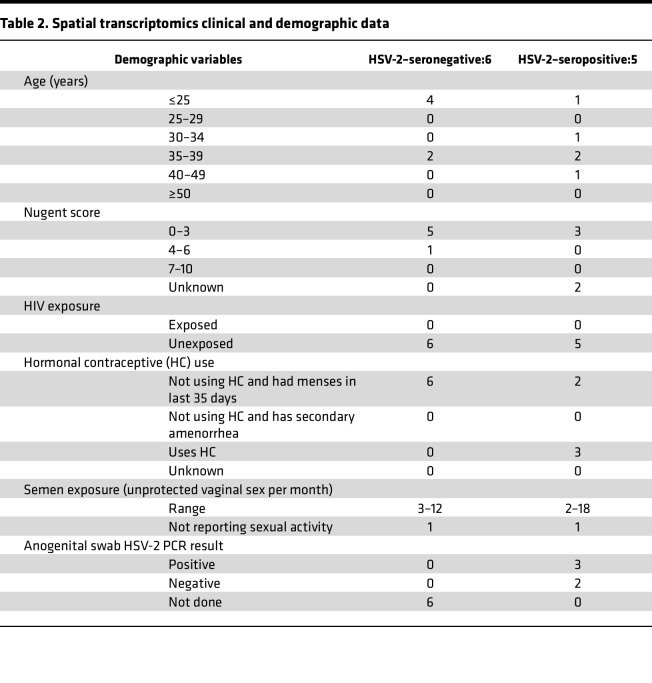
Spatial transcriptomics clinical and demographic data

## Version 1 11/18/2025

In-Press Preview

## Version 2 01/09/2026

Electronic publication
