# Supplementary material for: Genital herpes shedding episodes associate with altered spatial organization and activation of mucosal immune cells
Source: JCI Insight. 2025 Nov 18;11(1):e197491. doi: 10.1172/jci.insight.197491 (PMC12890532; doi:10.1172/jci.insight.197491)
Supplement: Supplemental data [file jciinsight-11-197491-s156.pdf]

Supplemental Methods

Participants, samples, and data collection

Samples for this analysis were provided by a subset of participants from the Kinga Study (Clinicaltrials.gov ID# NCT03701802). In total, the Kinga Study enrolled 406 heterosexual Kenyan couples to study how sexually transmitted infections and other exposures affect genital tract and systemic immune responses. Of the 406 couples enrolled, this analysis includes N=135 HSV-2 seronegative and N=97 HSV-2 seropositive individuals identified as female at birth (Table 1). Inclusion criteria for this analysis included those with a conclusive (positive or negative) HSV-2 serologic test result and a negative HIV-1/2 serologic test. Positive calls for the HerpeSelect-2 EIA (Focus Technologies, Cypress CA) assay used an index value cut-off of 3.4 (and a negative value below 1.8) to improve test specificity (1-4). Additionally, only samples that were provided by participants at their enrollment visit were analyzed here.

Eight sample types were requested (in addition to diagnostic clinical samples) from each Kinga Study participant for immune characterization studies. These include two 3-mm VT tissue biopsies and two 3-mm CX tissue biopsies. Each biopsy was cryopreserved with one VT and one CX biopsy cryopreserved in optimal cutting temperature (OCT) and used for cryosectioning and immunofluorescent imaging and/or spatial transcriptomics analysis, and one VT and one CX biopsy cryopreserved for high-parameter flow cytometry analysis. Fractionated PBMC samples were also collected and cryopreserved for high-parameter flow cytometry analysis. CVT fluid was collected by menstrual cup (Softcup®) and, along with serum samples, analyzed for the presence and concentration of soluble immune factors. Additionally, an anogenital swab was collected at the study visit for PCR testing to detect active HSV-2 viral shedding. Using a Dacron

swab, the anogenital region was sampled, which includes the cervix and vagina, labia majora and minora and perineum/perianal region. Sample collection and clinical testing procedures for the Kinga Study, including HIV-1/2 and HSV-2 serology testing methods, were performed in the same manner as previously described (1-3, 5). For flow cytometry analyses, 124 HSV-2 seronegative and 85 HSV-2 seropositive individuals provided a VT tissue biopsy, an overlapping but unique set of 124 HSV-2 seronegative and 85 HSV-2 seropositive individuals provided a CX tissue biopsy, and 130 HSV-2 seronegative and 92 HSV-2 seropositive provided a PBMC sample. Soluble immune factors were measured from cervicovaginal tract (CVT) secretions from 114 HSV-2 seronegative and 82 HSV-2 seropositive individuals and in serum samples from 134 HSV-2 seronegative and 97 HSV-2 seropositive individuals. We report image analysis results from a subset of participants selected as unexposed to HIV and BV negative (Nugent Score 0-3) (CX: HSV-2 seronegative N=44, HSV-2 seropositive N=19; VT: HSV-2 seronegative N=55, HSV-2 seropositive N=24). The spatial transcriptomic analysis consisted of a smaller subset of individuals described below in the spatial transcriptomics section.

Immunofluorescent microscopy analysis

A subset of participants in this analysis provided a VT or CX sample that was sectioned and analyzed for the quantity and spatial arrangement of $CD3^{+}$, $CD4^{+}$, and $CCR5^{+}$ cells. This subset includes only participants who were not exposed to HIV and had no BV (a Nugent score of 0-3). Biopsies were collected by clinicians at the study site using Tischler forceps at the lateral VT wall and/or the ectocervical os, embedded in OCT compound, and cryopreserved on dry ice. Laboratory analysis, including tissue processing, staining, imaging, and other assay components, including tissue section image analysis, was done exactly as described previously (5). The codes

and an extended description of the GUI used to analyze immunofluorescent images in MATLAB (R2022b) can be found at github.com/FredHutch/Kinga_Study_BV_MacLean (5).

High-parameter flow cytometry analysis

Participants also provided VT and CX biopsies cryopreserved for high-parameter flow cytometry analysis. Study site clinicians collected these biopsies using Tischler biopsies at the VT wall or cervical os. Biopsies were placed in cryovial with 4% fetal bovine serum at 4°C, transported briefly to the lab, then cryopreserved overnight at -80°C in dimethyl sulfoxide (DMSO), and transferred to liquid nitrogen for long-term storage as described previously (6). PBMCs were also collected, processed, and cryopreserved in DMSO. All processing for genital tissues and PBMC samples was done previously as described in detail (5). Samples were then shipped to the University of Washington for further analysis. Cryopreserved tissue biopsies and PBMC samples were quickly thawed, processed, and stained with fluorescently labeled antibodies as previously described (5). After isolation, cells were incubated with UV Blue Live/Dead reagent for 30 minutes at room temperature and then stained with the antibodies and protocol previously described (5). Analysis was performed using Flowjo software. Gating analysis was also replicated as described by Maclean et al. 2025, with a 25 cell minimum required to proceed to any downstream gate. The proportion of each cell type was then calculated by dividing the count of a phenotype by the count of its parent gate.

Cytokine and chemokine analysis

A menstrual cup inserted into the vaginal canal of participants who were not actively menstruating was used to collect CVT fluid for analysis of cervicovaginal soluble immune

factors. Serum samples were also collected to analyze circulating soluble immune factors. CVT fluid and serum samples were processed, cryopreserved, and shipped to the University of Washington and then to Eve Technologies (Calgary, Alberta, Canada). The detection and quantification of chemokines and cytokines were done using the Human Cytokine Array/Chemokine Array 71-403 Plex Panel (Eve Technologies, HD71). All collection and processing were done as described previously (5).

Spatial Transcriptomics Sampling and Processing

In addition to previously described clinical testing, a subset of HSV-2 seropositive individuals provided an anogenital swab at the same time as biopsy collection for the detection of viral DNA via PCR testing previously described by Jerome *et al.* (7). PCR was performed on a total of 85 anogenital swabs at the University of Washington Virology Lab. N=12 swabs were positive for HSV-2, indicating that these participants were actively shedding HSV-2 virus at the same time as biopsy collection. A subset of 3 individuals with a positive HSV-2 PCR result, 2 individuals HSV-2 seropositive but with a negative HSV-2 PCR result, and 6 HSV-2 seronegative participants provided fresh VT biopsies, which were cryopreserved in OCT and included in the spatial transcriptomics analysis. Of the five HSV-2 seropositive participants included here, only 1 reported having sores in the last three months. All 5 HSV-2 seropositive participants showed no signs of active genital herpes during their clinical exam at the visit.

Fresh frozen VT biopsies from this subset of participants were prepared according to the 10x Genomics tissue preparation protocol (CG000579, Rev C). OCT blocks were cryosectioned at 10μm, adhered to equilibrated Xenium slides, and stored at -80°C for later processing. Tissue sections were then fixed in paraformaldehyde and permeabilized following the protocol

CG000581 Rev C. Probe hybridization of 377 genes in the Xenium Human Multi-Tissue and Cancer panel (10x Genomics, 2023), ligation, and amplification were performed as instructed in CG000582 Rev E, followed by nuclei staining and removal of background fluorescence. Imaging and decoding of fluorescent probe optical signatures were performed onboard the 10x Genomics Xenium platform (software version 1.7.6.0 and analysis version xenium-1.7.1.0) with additional analysis to detect the abundance and localization of mRNA transcripts.

Xenium Analysis

Data preprocessing and cell segmentation

Cell segmentation was initially performed with 10x Xenium onboard cell segmentation (10x Genomics). DAPI-stained nuclei from the DAPI morphology image were segmented, and boundaries were consolidated to form nonoverlapping objects. Next, we used Proseg v1.1.8 (8) cell segmentation algorithm, which uses cell morphologies identified as described above, plus the spatial distribution of transcripts to determine cell boundaries. Samples were then processed with Xenium Ranger v 3.0.1.

Quality filtering and data preprocessing

Data was loaded and analyzed using R version 4.3.1. For each sample, Xenium generated an output file of transcript information, including x and y coordinates, corresponding gene, assigned cell and/or nucleus, and quality score. Low-quality transcripts (quality value (QV) < 20) and transcripts corresponding to blank probes were removed. Seurat v5 (9) was used to perform further quality filtering and visualization on gene expression data. A Seurat object was created for each sample, containing probe counts, metadata, centroids, segmentations, and molecules.

Cells that contained ≥10 transcripts corresponding to ≥5 unique genes were retained. Individual samples were normalized individually, and a single merged Seurat object containing a total of 142,903 cells was created for all samples for scaling and PCA. To account for interpatient sample-specific variability, samples were integrated using Seurat RPCA integration. The integrated object was used to generate UMAP and clustering using Seurat FindClusters with a resolution of 0.5.

Cell type identification

To annotate cell clusters, SingleR v2.4.1 (10)was employed to compare against 6 different references encompassing both tissue and immune cell atlases available through the SingleR celldex v1.12.0 package (Human Primary Cell Atlas, Database of Immune Cell Expression, ENCODE Blueprint data, ImmGen 28 cell, Monaco Immune data, Mouse Cell Atlas (converted to human genes)). 12 main clusters were identified and annotated. The T cell and macrophage/DC clusters were subsetted and regressed for genes associated with their spatial proximity, such as epithelial and fibroblast genes, and sub-clustered to identify T cell and myeloid subsets.

Gene expression

For analysis, HSV+ samples were defined as Shed+ or Shed- as described above. Seurat's FindMarkers function was used to find differentially expressed genes for each subtype between Shed+ and Shed- samples (LFC>1 and padj<0.05). Due to the probe-count-based approach of the Xenium platform, which does not reflect true gene counts, we considered a cell positive for a gene if it contained 1 or more normalized counts, and calculated the percentage of cells positive for a gene as a comparator. Heatmaps were generated using ggplot2 v3.5.1(11).

Identification of tissue compartments

For spatial analyses, individual samples were resubsetted from the merged Seurat object. To discriminate the localization of immune cells between the epithelium and lamina propria, Seurat's BuildNicheAssay was used on each sample with niches, k = 2, and neighbors, k = 5, to define 2 niches with enough definition to also identify the pockets of lamina propria which bud into the epithelial layer. Cells were designated to either niche, and subsequent analysis compared Shed+ and Shed- samples by niche. To calculate the proportion of a specific cell type, the number of cells was divided by the total number of cells in each niche, per sample. This was done to account for sampling differences of the tissue sections, which had differing numbers of cells and skewing due to the amount of epithelial vs lamina propria tissue captured.

Cell distances

To calculate the distance of cells from the border between the two niches, cell coordinates were extracted for each sample, then for each cell, the nearest inner epithelial cell was identified using the RANN package v2.6.1 (12), the distance was calculated, and each cell was assigned a distance value (µm).

Density

To calculate density, cell centroids were extracted for each sample and placed into 40 µm bins based on their coordinates. The number of cells of interest was counted for each bin, and bin indices were converted to spatial positions. The density was calculated as the number of cells per bin/area of the bin, and averaged to give cells per um^2. Due to the small sample size, statistical

significance between groups was assessed using a non-parametric, two-sided permutation test (n = 10,000 permutations), comparing the difference in means.

Co-localization

To determine which cells were in direct contact, a cut-off of 20 μm was used, and cells within that radius of a cell of interest were determined to be a "near" cell. A 20 μm cut-off was selected as lymphocytes are typically 10 μm in diameter, and macrophages are 20 μm in diameter, and the distance used is between cell centroids (the center of the cell). We therefore reasoned and confirmed that a distance of 20 μm between centroids would capture direct neighboring cells only. A previous study also found that 20 μm was robust in identifying cell-cell interactions (13). Cell centroids were extracted and nearest neighbors calculated using frNN from the dbscan package v 1.1-12 (14), with a neighborhood radius of 20. For each cell type of interest, the cell indices of interest were extracted along with the indices of the nearby cells. Cells that were identified as nearby cells were added to the metadata as "near" the cell of interest. To calculate the frequency of cell interactions in each niche, the number of specific cell interactions, classified as *subset of interest* *near_cell of interest* was divided by the total cells in each niche, per sample.

References.

1. Golden MR, Ashley-Morrow R, Swenson P, Hogrefe WR, Handsfield HH, and Wald A. Herpes simplex virus type 2 (HSV-2) Western blot confirmatory testing among men testing positive for HSV-2 using the focus enzyme-linked immunosorbent assay in a sexually transmitted disease clinic. *Sex Transm Dis.* 2005;32(12):771-7.
2. Laeyendecker O, Henson C, Gray RH, Nguyen RH, Horne BJ, Wawer MJ, et al. Performance of a commercial, type-specific enzyme-linked immunosorbent assay for detection of herpes simplex virus type 2-specific antibodies in Ugandans. *J Clin Microbiol.* 2004;42(4):1794-6.
3. Gamiel JL, Tobian AA, Laeyendecker OB, Reynolds SJ, Morrow RA, Serwadda D, et al. Improved performance of enzyme-linked immunosorbent assays and the effect of human immunodeficiency virus coinfection on the serologic detection of herpes simplex virus type 2 in Rakai, Uganda. *Clin Vaccine Immunol.* 2008;15(5):888-90.
4. Ashley-Morrow R, Nollkamper J, Robinson NJ, Bishop N, and Smith J. Performance of focus ELISA tests for herpes simplex virus type 1 (HSV-1) and HSV-2 antibodies among women in ten diverse geographical locations. *Clin Microbiol Infect.* 2004;10(6):530-6.
5. MacLean F, Tsegaye AT, Graham JB, Swarts JL, Vick SC, Potchen NB, et al. Bacterial vaginosis associates with dysfunctional T cells and altered soluble immune factors in the cervicovaginal tract. *J Clin Invest.* 2025.
6. Hughes SM, Ferre AL, Yandura SE, Shetler C, Baker CAR, Calienes F, et al. Cryopreservation of human mucosal tissues. *PLoS One.* 2018;13(7):e0200653.

7. Jerome KR, Huang ML, Wald A, Selke S, and Corey L. Quantitative stability of DNA after extended storage of clinical specimens as determined by real-time PCR. *J Clin Microbiol.* 2002;40(7):2609-11.

8. Jones DC, Elz AE, Hadadianpour A, Ryu H, Glass DR, and Newell EW. Cell simulation as cell segmentation. *Nat Methods.* 2025.

9. Hao Y, Stuart T, Kowalski MH, Choudhary S, Hoffman P, Hartman A, et al. Dictionary learning for integrative, multimodal and scalable single-cell analysis. *Nat Biotechnol.* 2024;42(2):293-304.

10. Aran D, Looney AP, Liu L, Wu E, Fong V, Hsu A, et al. Reference-based analysis of lung single-cell sequencing reveals a transitional profibrotic macrophage. *Nat Immunol.* 2019;20(2):163-72.

11. Wickham H. *Use R!,*. Cham: Springer International Publishing : Imprint: Springer,; 2016:1 online resource (XVI, 260 pages 32 illustrations, 140 illustrations in color.

12. Mount GJaSEKaSAaD. RANN: Fast Nearest Neighbour Search (Wraps ANN Library) Using L2 Metric. https://github.com/jefferislab/RANN.

13. Alon S, Goodwin DR, Sinha A, Wassie AT, Chen F, Daugharthy ER, et al. Expansion sequencing: Spatially precise in situ transcriptomics in intact biological systems. *Science.* 2021;371(6528).

14. Hahsler M, Piekenbrock M, and Doran D. dbscan: Fast Density-Based Clustering with R. *Journal of Statistical Software.* 2019;91(1):1 - 30.

Supplemental Acknowledgments

Kinga Study Team:

University of Washington: International Clinical Research Center

Jairam R Lingappa (co-Principal Investigator and Protocol Chair), Justice Quame-Amaglo (study coordinator), Harald Haugen, Elena Rechkina (Laboratory Director), Daphne Hamilton, Matthew Ikuma, Marie Bauer, Zarna Marfatia, Kathy Thomas, Corinne Mar, Adino Tesfahun Tsegaye, Ayumi Saito

Fred Hutchinson Cancer Center:

Jennifer Lund (co-Principal Investigator), Sarah Vick, Finn MacLean, Jessica Graham, Jessica Swarts, Nicole Potchen, Irene Cruz Talavera, Lakshmi Warrier, Laura Pattacini, Paula Culver

Kenya Medical Research Institute: Thika Partners in Health Research and Development (and Jomo Kenyatta University [JKUAT])

Nelly R. Mugo (Site Principal Investigator), Kenneth Ngure (Site Investigator), Catherine Kiptinness (site coordinator), Bhavna H. Chohan (Site Laboratory Director), Nina Akelo, Stephen Gakuo, Elizabeth Irungu, Marion Kiguoya, Edith Kimani, Eric Koome, Solomon Maina, Linet Makena, Sarah Mbaire, Murugi Micheni, Peter Michira, Peter Mogere, Richard Momanyi, Edwin Mugo, Caroline Senoga, Mary Kibatha, Jelioth Muthoni, Euticus Mwangi, Philip Mwangi, Margaret Mwangi, Charles Mwangi, Stanley Mugambi Ndwiga, Peter Mwenda, Grace Ndung'u, Faith Njagi, Zakaria Njau, Irene Njeru, John Njoroge, Esther Njoroge, John Okumu, Lynda Oluoch, Judith Achieng Omungo, Snaida Ongachi, Dennis Wanyonyi, Everlyne Okong'o, Agnes Ndirangu

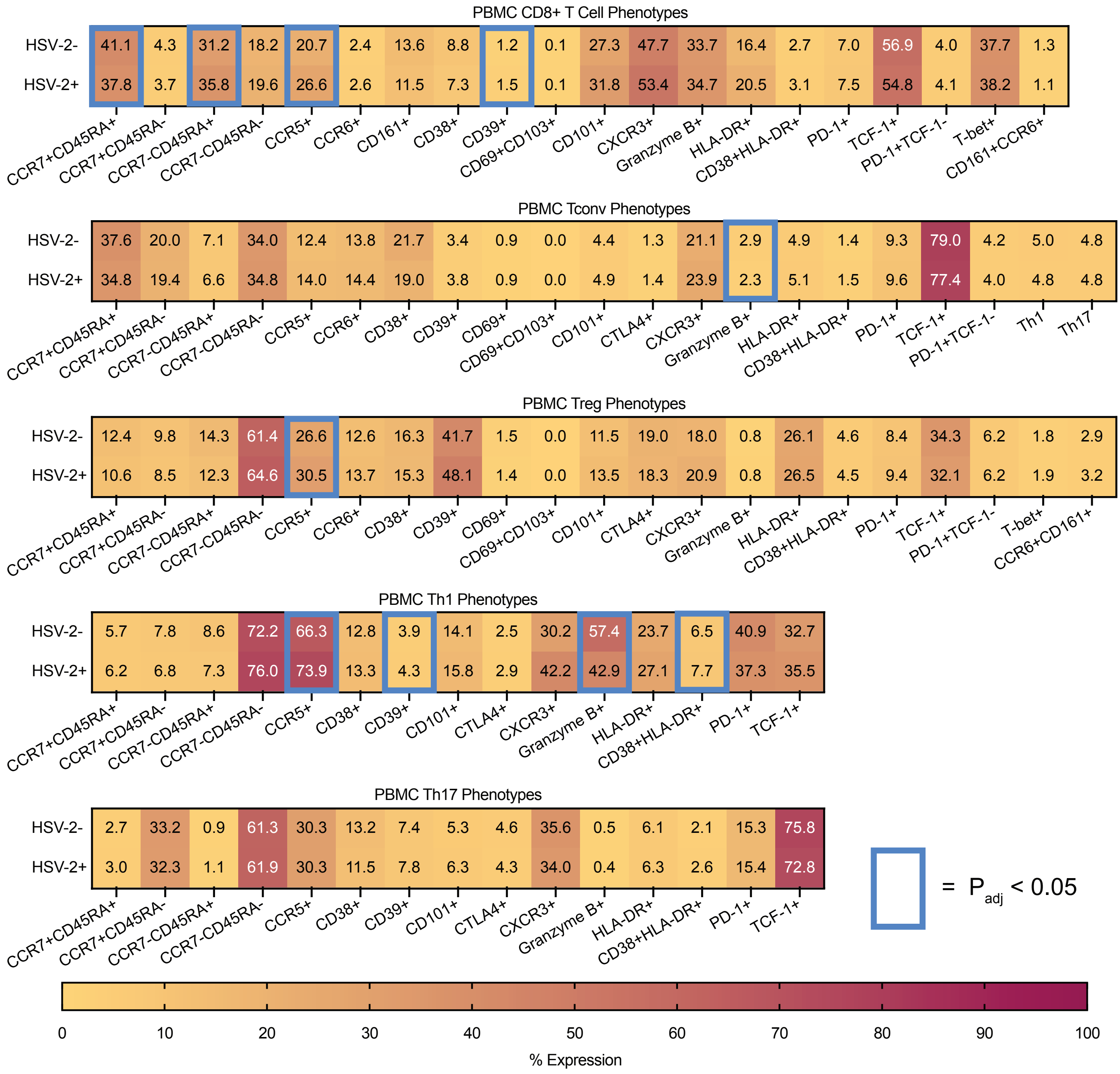

Supplemental Figure 1 - Circulating T cell phenotypes associated with HSV-2 seropositivity
Heatmaps showing the frequency of CD8+ T cell, Tconv, Treg, Th1, and Th17 phenotypes in PBMC samples from HSV-2 seropositive and seronegative individuals. Significant results comparing HSV-2 seropositive vs seronegative, using an adjusted rank regression model that adjusted for hormonal contraceptive use and age, are boxed in blue. N=130 HSV-2 seronegative and N=92 HSV-2 seropositive.

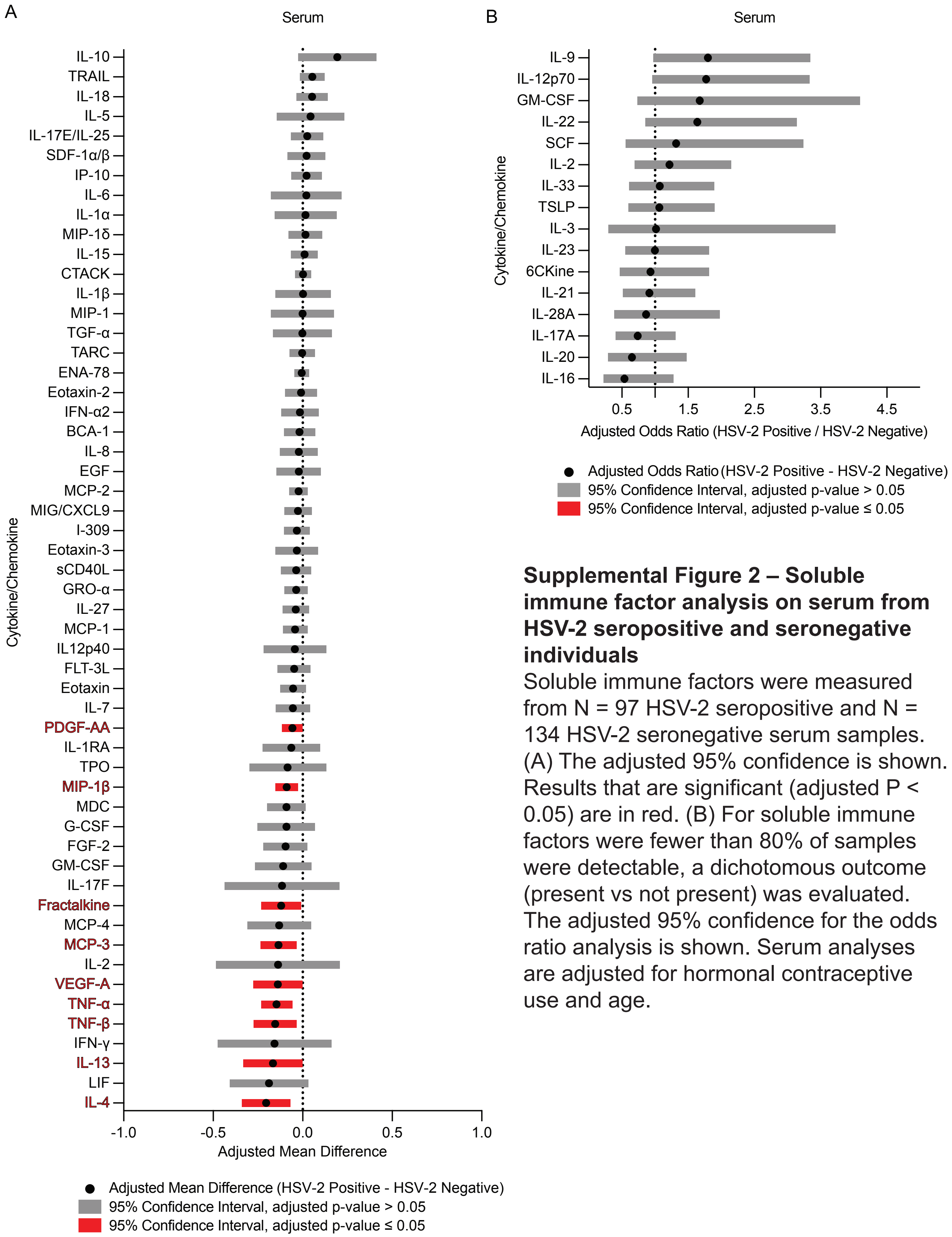

Supplemental Figure 2 – Soluble immune factor analysis on serum from HSV-2 seropositive and seronegative individuals

Soluble immune factors were measured from N = 97 HSV-2 seropositive and N = 134 HSV-2 seronegative serum samples. (A) The adjusted 95% confidence is shown. Results that are significant (adjusted P < 0.05) are in red. (B) For soluble immune factors were fewer than 80% of samples were detectable, a dichotomous outcome (present vs not present) was evaluated. The adjusted 95% confidence for the odds ratio analysis is shown. Serum analyses are adjusted for hormonal contraceptive use and age.

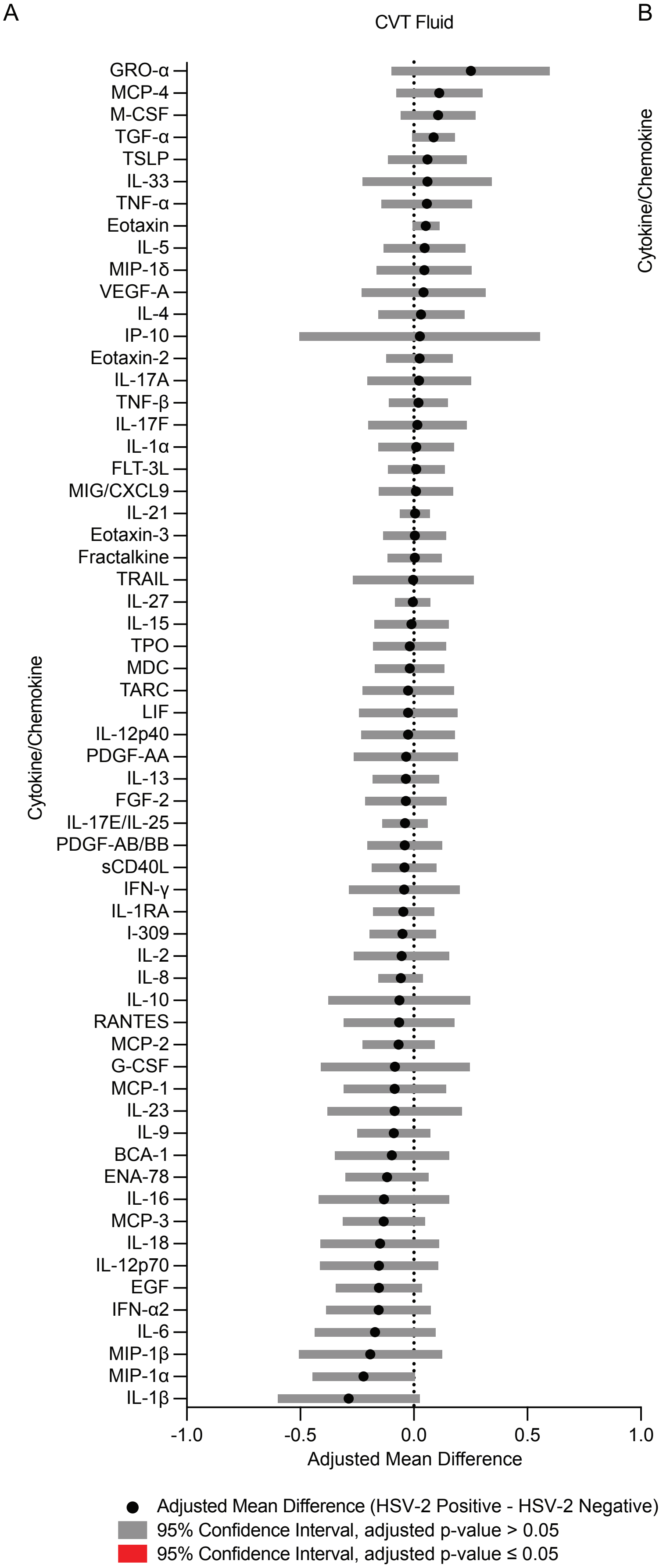

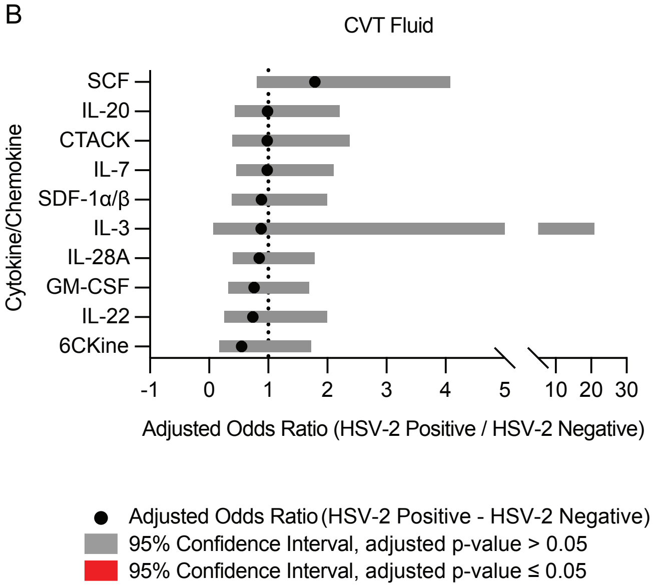

Supplemental Figure 3 – Soluble immune factor analysis on CVT fluid from HSV-2 seropositive and seronegative individuals

Soluble immune factors were measured from N = 82 HSV-2 seropositive and N = 114 HSV-2 seronegative cervicovaginal fluid samples. (A) The adjusted 95% confidence is shown. Results that are significant (adjusted P < 0.05) are in red. (B) For soluble immune factors were fewer than 80% of samples were detectable, a dichotomous outcome (present vs not present) was evaluated. The adjusted 95% confidence for the odds ratio analysis is shown. Cervicovaginal fluid samples comparisons are adjusted for hormonal contraceptive use, age, semen exposure, HIV exposure, and bacterial vaginosis via Nugent score.

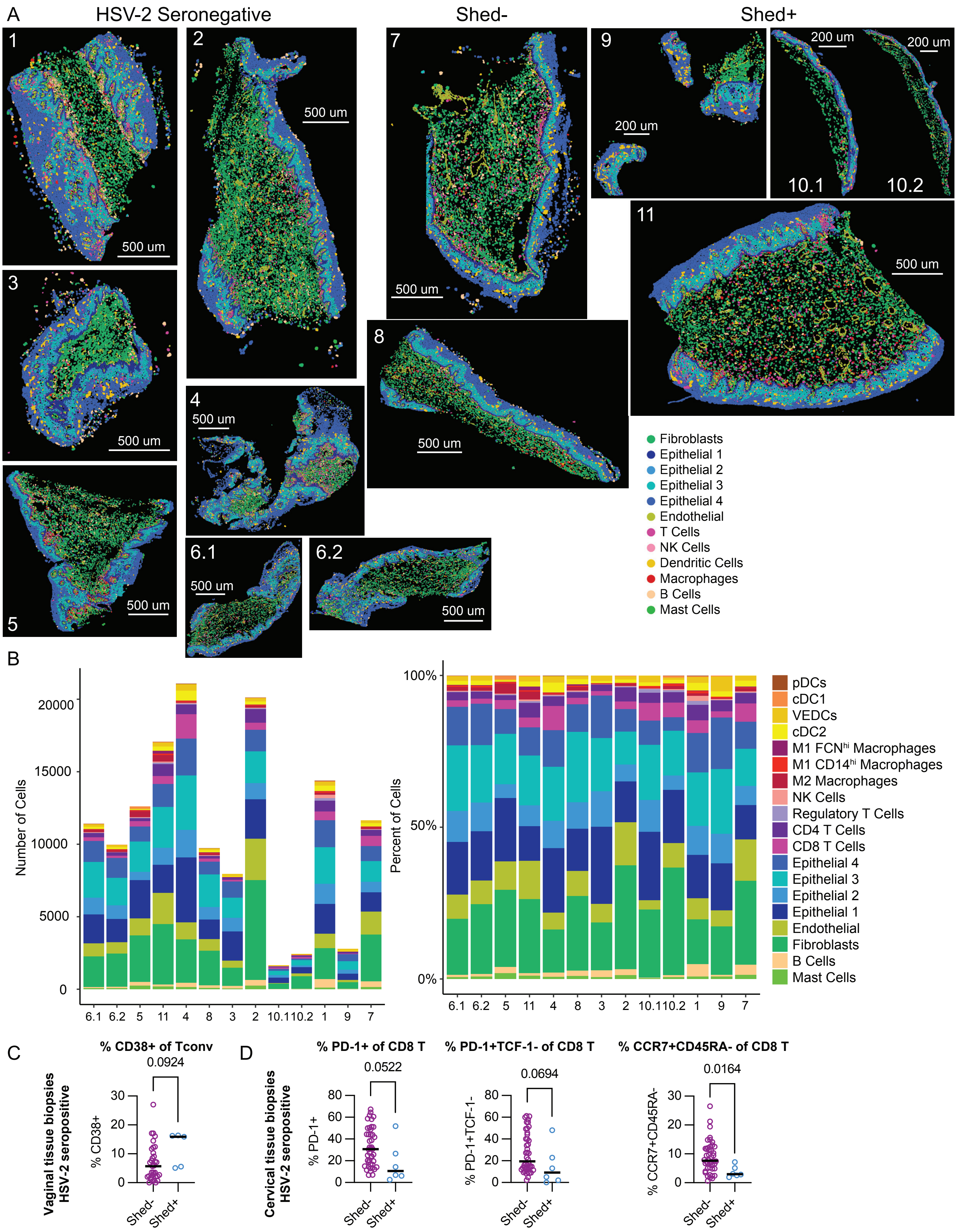
A
HSV-2 Seronegative
Shed-
Shed+
1
2
3
4
5
6.1
6.2
7
8
9
10.1
10.2
11
500 um
200 um
Fibroblasts
Epithelial 1
Epithelial 2
Epithelial 3
Epithelial 4
Endothelial
T Cells
NK Cells
Dendritic Cells
Macrophages
B Cells
Mast Cells
B
Number of Cells
Percent of Cells
pDCs
cDC1
VEDCs
cDC2
M1 FCN^{hi} Macrophages
M1 CD14^{hi} Macrophages
M2 Macrophages
NK Cells
Regulatory T Cells
CD4 T Cells
CD8 T Cells
Epithelial 4
Epithelial 3
Epithelial 2
Epithelial 1
Endothelial
Fibroblasts
B Cells
Mast Cells
C
Vaginal tissue biopsies HSV-2 seropositive
% CD38+ of Tconv
0.0924
% CD38+
D
Cervical tissue biopsies HSV-2 seropositive
% PD-1+ of CD8 T
0.0522
% PD-1+
% PD-1+TCF-1- of CD8 T
0.0694
% PD-1+TCF-1-
% CCR7+CD45RA- of CD8 T
0.0164
% CCR7+CD45RA-
Shed-
Shed+

Supplemental Figure 4 – Spatial identity and phenotype of cervicovaginal immune cells based on HSV-2 shedding and HSV-2 serostatus

11 samples were included in the spatial transcriptomics analysis. This includes 6 HSV-2 seronegative, 2 HSV-2 seropositive, anogenital HSV-2 PCR negative, and 3 HSV-2 seropositive, anogenital HSV-2 PCR positive (A). (B) The number of cells captured per sample and the proportion of each cell subset per sample is depicted graphically. The biopsy size and number of cells acquired from each sample was variable, but each cell population was represented in each tissue, and the proportion of the cell types was relatively stable. (C and D) T cell phenotypes in vaginal and cervical tissue biopsies from HSV-2-seropositive individuals only were assessed by flow cytometry as in Figure 2. Comparisons were made based on anogenital HSV-2 shedding status at the time of biopsy collection. (C) The only statistical trend ($p<0.1$) found in vaginal tissue biopsies was in the % of Tconv expressing CD38. (D) The only statistically significant difference ($P<0.05$) or statistical trend ($p<0.1$) found in cervical tissue biopsies was in the % of PD-1^+, PD-1^+TCF-1-, or $CCR7^+CD45RA^-$ among $CD8^+$ T cells. Mann-Whitney tests with no adjustments were used to determine statistical significance in C and D.

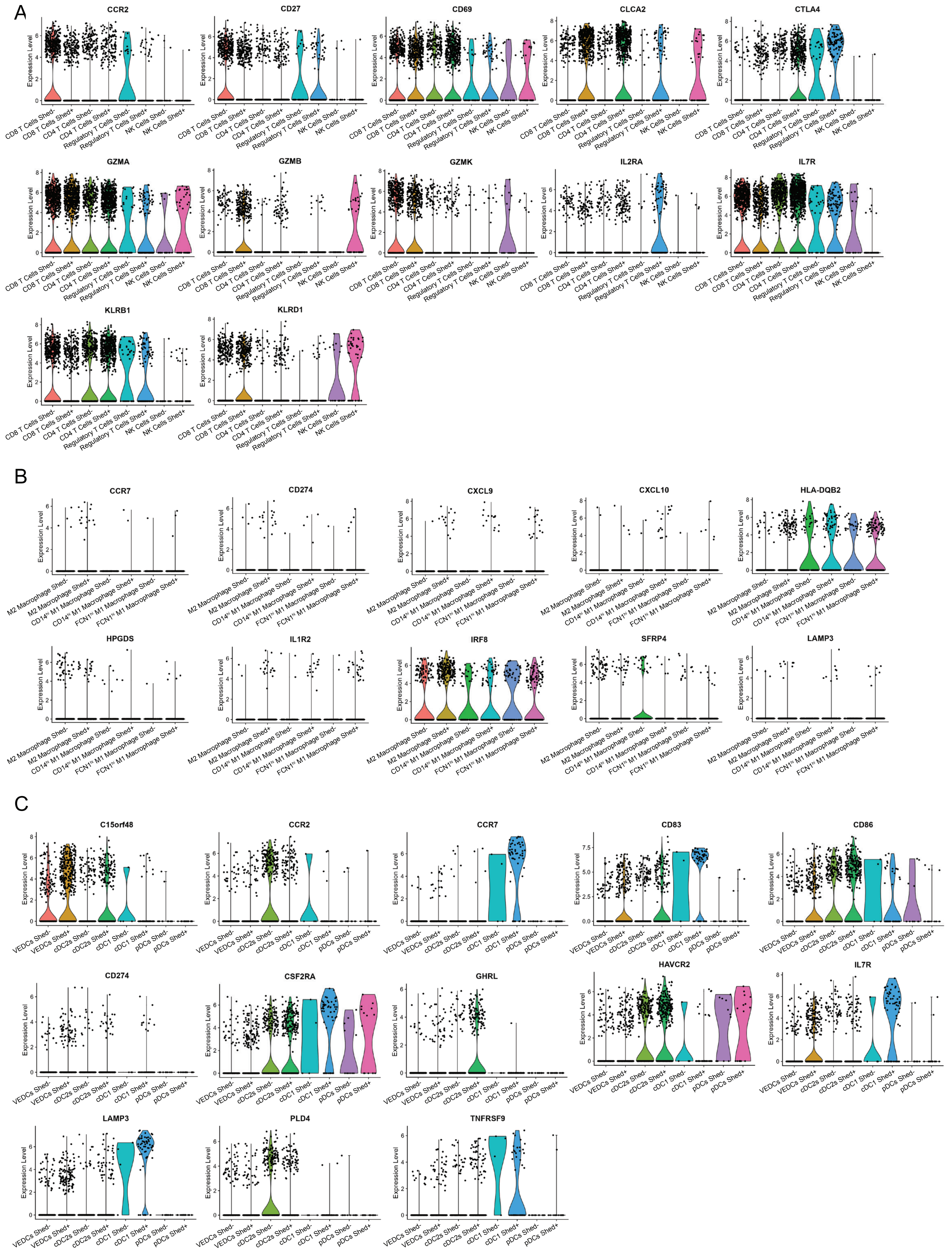
A
CCR2
CD27
CD69
CLCA2
CTLA4
GZMA
GZMB
GZMK
IL2RA
IL7R
KLRB1
KLRD1
Expression Level
CD8 T Cells Shed-
CD8 T Cells Shed+
CD4 T Cells Shed-
CD4 T Cells Shed+
Regulatory T Cells Shed-
Regulatory T Cells Shed+
NK Cells Shed-
NK Cells Shed+
B
CCR7
CD274
CXCL9
CXCL10
HLA-DQB2
HPGDS
IL1R2
IRF8
SFRP4
LAMP3
M2 Macrophage Shed-
M2 Macrophage Shed+
CD14hi M1 Macrophage Shed-
CD14hi M1 Macrophage Shed+
FCN1hi M1 Macrophage Shed-
FCN1hi M1 Macrophage Shed+
C
C15orf48
CCR2
CCR7
CD83
CD86
CD274
CSF2RA
GHRL
HAVCR2
IL7R
LAMP3
PLD4
TNFRSF9
VEDCs Shed-
VEDCs Shed+
cDC2s Shed-
cDC2s Shed+
cDC1 Shed-
cDC1 Shed+
pDCs Shed-
pDCs Shed+

Supplemental Figure 5 – Gene expression in immune cell subsets from HSV-2 Shed- and Shed+ individuals
Violin plots showing the scaled expression of genes identified as differentially expressed between Shed+ and Shed- in (A) T cell subsets and NK cells, (B) macrophage subsets, and (C) dendritic cell subsets.

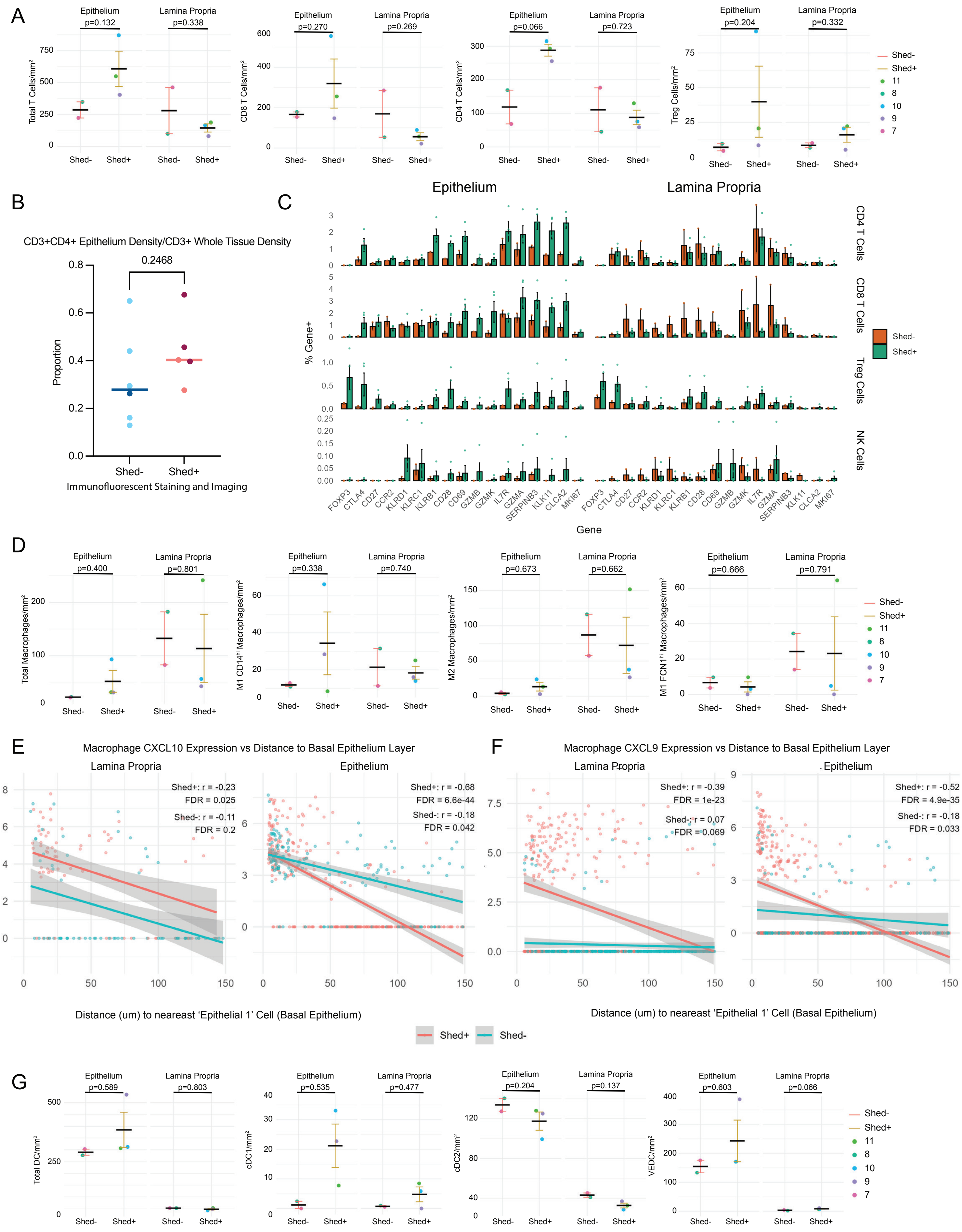
A
Epithelium
Lamina Propria
Total T Cells/mm²
CD8 T Cells/mm²
CD4 T Cells/mm²
Treg Cells/mm²
Shed-
Shed+
B
CD3+CD4+ Epithelium Density/CD3+ Whole Tissue Density
0.2468
Proportion
Immunofluorescent Staining and Imaging
C
% Gene+
CD4 T Cells
CD8 T Cells
Treg Cells
NK Cells
Gene
D
Total Macrophages/mm²
M1 CD14hi Macrophages/mm²
M2 Macrophages/mm²
M1 FCN1hi Macrophages/mm²
E
Macrophage CXCL10 Expression vs Distance to Basal Epithelium Layer
F
Macrophage CXCL9 Expression vs Distance to Basal Epithelium Layer
Distance (um) to nearest 'Epithelial 1' Cell (Basal Epithelium)
G
Total DC/mm²
cDC1/mm²
cDC2/mm²
VEDC/mm²

Supplemental Figure 6 – Tissue location of immune cell subsets varies based on HSV-2 shedding status

(A) The total T cell density in each tissue compartment, separated by Shed- vs Shed+, calculated via spatial transcriptomics. (B) The $CD3^{+}CD4^{+}$ density in the epithelium divided by the total $CD3^{+}$ density in the tissue, as measured by immunofluorescent staining and cellular imaging, to account for differences in total cellularity and tissue size across samples. Each dot represents an individual sample, the bar represents the median. Darker shaded dots correspond to the sections from the same samples as those used in the Xenium experiment. N Shed- = 6, N Shed+ = 5. Comparison using Mann-Whitney test, P value displayed. (C) Bar plot of total gene expression to genes shown in the heatmap of Figure 6F. (D) The total macrophage and subset density in each tissue compartment, comparing Shed- vs Shed+, calculated via spatial transcriptomics. (E) A correlation graph between macrophage CXCL10 expression and distance to the nearest inner/basal epithelial cell stratified by Shed- vs Shed+. (F) A correlation graph between macrophage CXCL9 expression and distance to the nearest inner/basal epithelial cell, stratified by Shed- vs Shed+. (G) The total dendritic cell and subset density in each tissue compartment, comparing Shed- vs Shed+, calculated via spatial transcriptomics. Comparisons for density made using a non-parametric permutation test and for correlation graphs using Pearson correlation coefficients within each niche and Shed group. Statistical significance of each chemokine correlation was assessed using Pearson’s correlation test and corrected for multiple comparisons using FDR method.

Supplemental Figure 7

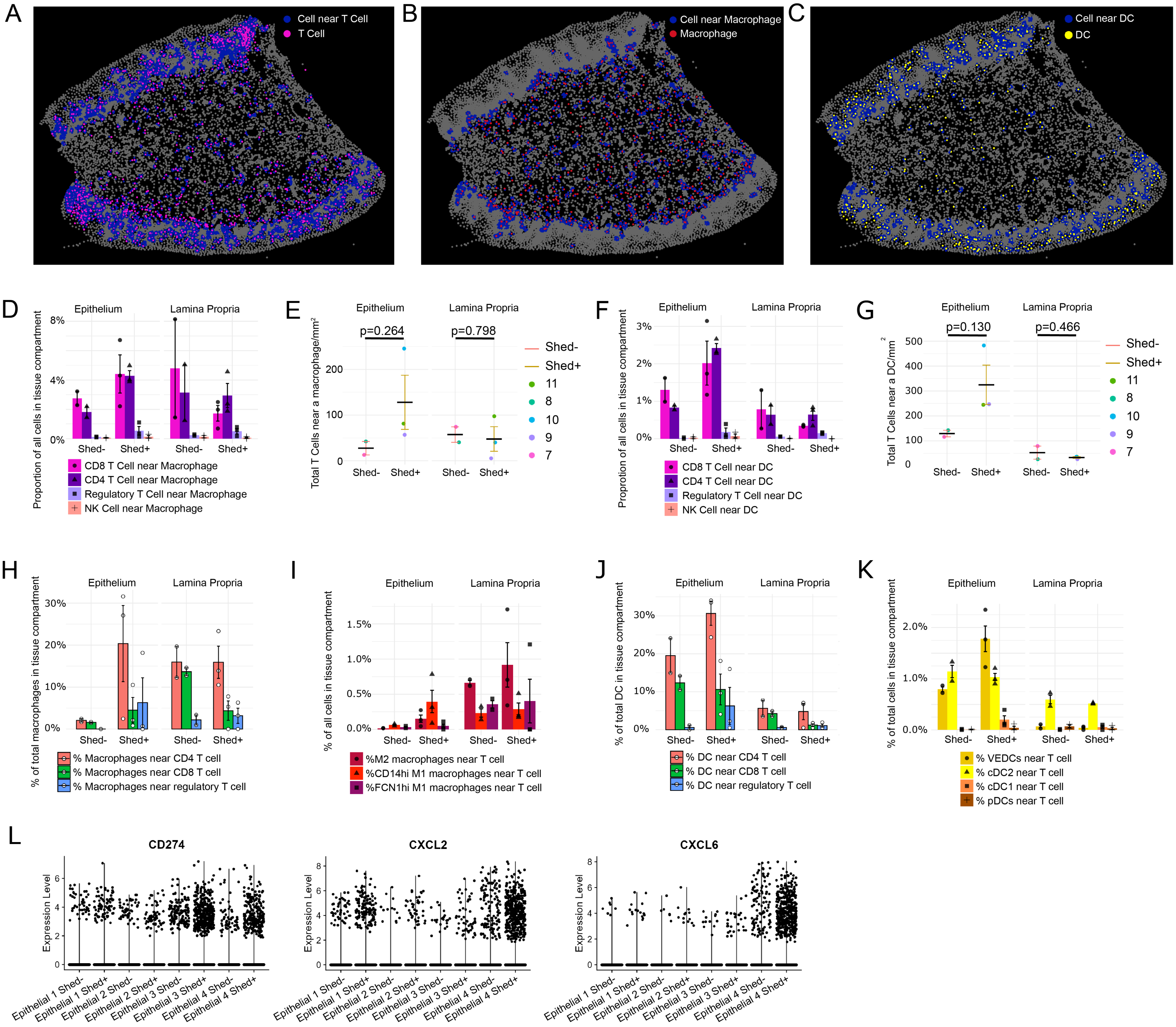

Supplemental Figure 7 – Immune cell organization in the vaginal epithelium and lamina propria in presence or absence of HSV-2 shedding episode

Representative image of (A) T cells (pink), (B) macrophages (red), or (C) dendritic cells (yellow), and any cell that colocalized near them (blue). (D) The proportion of T and NK cells near macrophages in either tissue compartment in Shed- and Shed+. (E) The density of T cells that colocalized with macrophages in Shed- vs Shed+. Each dot represents an individual sample. Comparison made using a non-parametric permutation test, P value displayed. (F) The proportion of T and NK cells near dendritic cells in either tissue compartment in Shed- and Shed+. (G) The density of T cells that colocalized with dendritic cells in Shed- vs Shed+. Each dot represents an individual sample. Comparison made using a non-parametric permutation test, P value displayed. (H) Bar plot showing the percentage of macrophages near T cell subsets in Shed- vs Shed+ by tissue compartment as a percentage of total macrophages in the tissue compartment. (I) Bar plot showing the percentage of macrophage subsets near a T cell in Shed- vs Shed+ by tissue compartment as a percentage of total cells in the tissue compartment. (J) Bar plot showing the percentage of dendritic cells near T cell subsets in Shed- vs Shed+ by tissue compartment as a percentage of total macrophages in the tissue compartment. (K) Bar plot showing the percentage of dendritic cell subsets near a T cell in Shed- vs Shed+ by tissue compartment as a percentage of total cells in the tissue compartment. For D-K, bars indicate mean and error bars show standard error of the mean. (L) Violin plots showing the scaled expression of genes identified as differentially expressed between Shed+ and Shed- in epithelial cell subsets.
